# Updated Progress on Polysaccharides with Anti-Diabetic Effects through the Regulation of Gut Microbiota: Sources, Mechanisms, and Structure–Activity Relationships

**DOI:** 10.3390/ph17040456

**Published:** 2024-04-02

**Authors:** Xiaoyu Zhang, Jia Wang, Tingting Zhang, Shuqin Li, Junyu Liu, Mingyue Li, Jingyang Lu, Min Zhang, Haixia Chen

**Affiliations:** 1 Tianjin Key Laboratory for Modern Drug Delivery and High-Efficiency, School of Pharmaceutical Science and Technology, Faculty of Medicine, Tianjin University, Tianjin 300072, China; 15153501767@163.com (X.Z.); jiawangcongcong@163.com (J.W.); 17622636510@163.com (T.Z.); lisq2013@sina.com (S.L.); junyuliu@tju.edu.cn (J.L.); lmy15357831056@163.com (M.L.); jyl10192022@163.com (J.L.); 2 China-Russia Agricultural Processing Joint Laboratory, Tianjin Agricultural University, Tianjin 300384, China; zhangmin@tjau.edu.cn; 3 State Key Laboratory of Nutrition and Safety, Tianjin University of Science & Technology, Tianjin 300457, China

**Keywords:** polysaccharides, anti-diabetic effects, the gut microbiota, mechanism, structure–activity relationship

## Abstract

Diabetes mellitus (DM) is a common chronic metabolic disease worldwide. The disturbance of the gut microbiota has a complex influence on the development of DM. Polysaccharides are one type of the most important natural components with anti-diabetic effects. Gut microbiota can participate in the fermentation of polysaccharides, and through this, polysaccharides regulate the gut microbiota and improve DM. This review begins by a summary of the sources, anti-diabetic effects and the gut microbiota regulation functions of natural polysaccharides. Then, the mechanisms of polysaccharides in regulating the gut microbiota to exert anti-diabetic effects and the structure–activity relationship are summarized. It is found that polysaccharides from plants, fungi, and marine organisms show great hypoglycemic activities and the gut microbiota regulation functions. The mechanisms mainly include repairing the gut burrier, reshaping gut microbiota composition, changing the metabolites, regulating anti-inflammatory activity and immune function, and regulating the signal pathways. Structural characteristics of polysaccharides, such as monosaccharide composition, molecular weight, and type of glycosidic linkage, show great influence on the anti-diabetic activity of polysaccharides. This review provides a reference for the exploration and development of the anti-diabetic effects of polysaccharides.

## 1. Introduction

Diabetes mellitus (DM) is a metabolic disease characterized by hyperglycemia, which is a typical and complex chronic metabolic disease worldwide. Generally, it is classified into type 1 DM (T1DM), type 2 DM (T2DM), gestational DM and specific types of diabetes due to other causes like monogenic diabetes syndromes [1]. T1DM is an autoimmune illness caused by the destruction of β cells, which typically results in a lack of insulin, accounting for 5–10% of the prevalence of DM [2]. About 90% of DM cases are T2DM, which is caused by a gradual decrease in β-cell insulin secretion [3].

The incidence of DM is rising as a result of the changing of lifestyles and living circumstances. DM is considered a global disease and more and more people suffer from it. According to a prediction of the International Diabetes Federation (IDF), there may be up to 700 million people living with DM by 2045. Persistent hyperglycemia can cause chronic damage to and dysfunction of numerous organs and tissues, such as eyes, kidneys, heart, blood vessels, and nerves [4,5]. For example, abnormal blood glucose metabolism is usually accompanied by blood lipid metabolism disorder [6]. Additionally, patients with DM have a notable increase in microvascular risk, which can lead to the development of hypertension [7]. What is more, DM may predispose toward Parkinson-like pathology [8,9]. These complications seriously endanger human health. Nowadays, the prevention and treatment of DM mainly focus on improvement in lifestyle and control of blood sugar by drugs. Although there are many drugs on the market that can be used to treat DM, like biguanides, acarbose, sulfonylureas and thiazolidinediones, these drugs have some limitations that cannot be ignored, such as side effects or high cost [10,11]. The development of lower-toxicity and more effective drugs is necessary.

Polysaccharides are carbohydrate macromolecules composed of at least more than 10 monosaccharides linked to each other by glycosidic bonds, and can be mainly found in plants, fungi, and marine organisms in nature. Numerous functions, including hypoglycemic, anti-inflammatory, immunomodulatory, neuromodulatory, and anticancer activities, have been demonstrated by studies on polysaccharides [12,13,14,15,16]. In addition, there are many advantages of polysaccharides, including good biodegradability and biocompatibility and few side effects [17,18]. Polysaccharides are utilized extensively in the food and pharmaceutical industries as emulsifiers, adjuvants for vaccines, and materials to treat oral diseases [19,20,21]. Therefore, polysaccharides can be regarded as a promising strategy for the prevention and treatment of DM.

The human gut is a crucial organ that is directly connected to the outside environment. The human the gut microbiota comprises trillions of microorganisms, including archaea, eukaryotes, viruses and bacteria at the domain level [22]. At the phylum level, it mainly consists of Firmicutes, Bacteroidetes, Actinobacteria, Proteobacteria, Verrucomicrobia and Fusobacteria [23,24,25]. They participate in nutrient metabolism, immune regulation and chemical modifications of dietary components, and the health of the host is closely related to the diversity and stability of the gut microbiota [26,27,28]. In normal conditions, the gut microbiota and its metabolites are maintained in a reasonably steady ecological balance with the host, but many factors can affect the composition and function of the gut microbiota such as dietary habits and hereditary and environmental factors [29]. The destruction of the gut microbiota may lead to many diseases, such as osteoporosis, obesity, T2DM, non-alcoholic fatty liver, and hypertension [30]. Research has found that a reduction in gut microbiota biodiversity and gut-associated metabolites contributes to heart failure [31]. The dysbiosis state of the gut microbiota and disruption in the energy metabolism contribute to the pathogenic mechanisms of neurodegeneration [32]. The modulation of gut microbiota biodiversity and gut-associated metabolites is associated with anti-diabetic activities [33,34,35,36].

The host cannot digest and absorb the majority of polysaccharides. In the human body, polysaccharides can interact with the gut microbiota and ferment into absorbable substances, mainly short-chain fatty acids (SCFAs) in the colon [37,38]. SCFAs consist of free fatty acids with two to five carbons, such as acetic acid, propanoic acid, and butyric acid, participating in signal transmission, nutrition provision and many other healthy protection functions in human body [39]. During these processes, polysaccharides are involved in the balance of the gut microbiota and host as a kind of prebiotic [40]. In turn, the gut microbiota can influence the host digestive efficiency by altering the bioavailability of polysaccharides [41].

In our earlier research, we discovered that mulberry leaf polysaccharides (MLPs) had a substantial negative link with the risk of obesity and might considerably lower the Firmicutes/Bacteroidetes (F/B) ratio, which may result in diminished energy absorption in mice [42]. Additionally, we discovered that *Lentinula edodes* soluble dietary fibers (LESDF) promoted Bacteroides production, indicating that LESDF was one of the dietary elements with an impact on the composition and relative abundance of the gut microbial community in vitro [43].

Nowadays, polysaccharides are highly valued by researchers in the field of medicine and health food and more and more polysaccharides have been discovered to prevent and treat DM. In addition, the gut microbiota is a hot topic in the management of DM. Although there are some reviews about the anti-diabetic effects and gut microbiota regulation function of polysaccharides, there is no comprehensive review about how polysaccharides exert anti-diabetic effects through the regulation of the gut microbiota and the related mechanisms. This article gives a summary of the relationship between polysaccharides and the gut microbiota in the prevention and treatment of DM, which may provide a reference for developing more effective anti-diabetic strategies.

## 2. Sources of Polysaccharides and Their Anti-Diabetic Effects

In the 21st century, scientific researchers paid more attention to natural products. Polysaccharides, natural compounds with low toxicity and multiple functions, can be regarded as lead compounds to be screened for developing new drugs and health foods [44]. Researchers discovered that the gut microbiota were linked to the onset of DM and played an important role in the maintenance of good health [23]. Although the human digestive system is capable of breaking down and absorbing certain polysaccharides like starch, there are polysaccharides that cannot be digested by the host because of high molecular weight and low bioavailability. They can enter the colon and interact with the gut microbiota, exerting dietary or medicinal effects [45,46]. For example, researchers found that polysaccharides from *Dendrobium officinale* were fermented and degraded into SCFAs in the large intestine and modulated bioactivities associated with the gut microbiota in rats [47].

**Figure 1:**
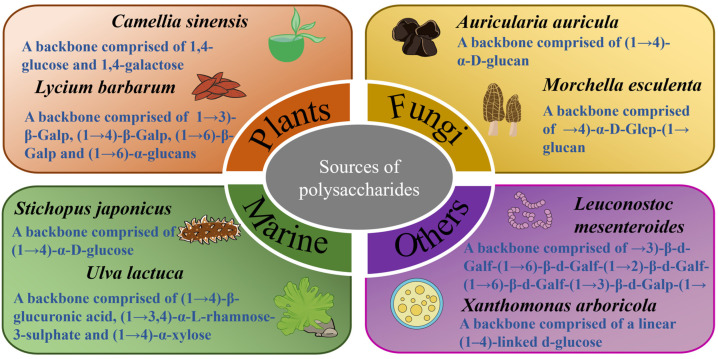
Sources of polysaccharides and the structures of some examples [48,49,50,51,52,53,54,55].

The sources of polysaccharides and the structure of some examples are shown in Figure 1. The anti-diabetic effects of polysaccharides from different sources and their regulation effects on the gut microbiota are summarized in the following.

### 2.1. Polysaccharides Extracted from Plants

Plants are one of the main sources of polysaccharides. Polysaccharides can be extracted from different kinds of plants like herbs, woody plants, and shrubs [56]. For example, *Camellia sinensis*, which is commonly called a tea tree, is a kind of shrub and the source of tea polysaccharides with famous anticancer and anti-diabetic effects in vitro or in vivo [57,58]. Various plant parts, including the leaves, stems, roots, and flowers, can also be used to extract polysaccharides. Polysaccharides extracted from the leaves and fruits of baobab (*Adansonia digitata* L.) have different molecular weights, solution viscosity-enhancing properties and other characteristics [59]. In our previous study, we found that polysaccharides from plants showed good anti-diabetic effects. Polysaccharides from *Camellia sinensis* showed α-glucosidase inhibitory capacity and hypoglycemic effect on L6 cells by promoting glucose uptake [60]. MLP treatment considerably improved metabolic diseases by lowering the Firmicutes/Bacteroidetes (F/B) ratio and increasing the amounts of Bifidobacterium, Lactobacillus, and Akkermansia, which were linked to the generation of butyric acid and/or propionic acid in mice [42].

Many other polysaccharides from various plants also showed anti-diabetic effects and the gut microbiota regulation function. The sources, structures, anti-diabetic effects and the gut microbiota regulation function of polysaccharides from plants are shown in Table 1.

Polysaccharides from plants show different gut microbiota regulation function. On the one hand, polysaccharides increase the diversity of probiotics in gut. Probiotics in the gut show positive effects on DM, such as decreasing lipid accumulation, ameliorating oxidative stress and inflammation, and increasing SCFAs in the gut [61]. Lactobacillus and Bifidobacterium are two main probiotics at the genus level and can even be used commercially [62,63]. Lactobacillus participates in glycolipid metabolism and produces SCFAs like lactic acid and acetic acid [64]. One study found that Lactobacillus could also reduce hepatic insulin resistance and liver deterioration in diabetic mice [65]. Bifidobacterium has great saccharolytic capability, plays an anti-inflammatory role, manages obesity, protects mucosal barrier integrity, and participates in the production of SCFAs in the gut [66,67].

Polysaccharides from kiwifruit (*Actinidia chinensis*), ginseng, and soy hull increase the abundance of Lactobacillus in mice or rats [68,69,70]. Polysaccharides from the tuberous root of *Ophiopogon japonicus* significantly enrich Bifidobacterium in mice [71,72]. On the other hand, polysaccharides regulate the gut microbiota by decreasing pathogenic bacteria. For example, the in vitro fermentation of human fecal microbiota showed that polysaccharides from *Polygonatum kingianum* decrease the abundance of Proteobacteria, which was positively correlated with dysbiosis of the gut microbiota and the occurrence of DM. It exhibited similar effects in diabetic rats [73,74].

### 2.2. Polysaccharides Extracted from Fungi

Fungi have been widely eaten and used by humans all over the world for thousands of years. Polysaccharides from fungi can be extracted from different parts like fruiting body, mycelium, or fermentation broth, and β-glucan accounts for the main structure of fungi polysaccharides [75,76]. Fungi polysaccharides possess many health-enhancing activities, such as immune modulation, fatigue relief, anti-aging and hypolipidemic activities in vivo or in vitro [77,78,79,80,81]. For example, researches revealed that oral β-1,3/1,6 glucan per day prevented the occurrence or reduced the severity of upper respiratory tract infection in older individuals [82]. High-purity yeast β-glucan induced innate immune regulation in gut mucosa, protecting against T1DM in mice [83]. *Grifola frondose* polysaccharides improved the disorder in lipid metabolism in high-fat diet (HFD)- and STZ-induced mice by increasing hepatic Acox1 expression and reducing the hepatic free fatty acid levels [84].

Polysaccharides from fungi can play an anti-diabetic role through modulation of the gut microbiota. The sources, structures, anti-diabetic effects and gut microbiota regulation function of polysaccharides from fungi are shown in Table 2.

Polysaccharides from fungi have anti-diabetic and the gut microbiota regulation functions in different respects. For example, *Grifola frondosa* polysaccharides improved the diversity and composition of the gut microbiota in HFD-induced diabetic mice [85]. Polysaccharides from *Agaricus bisporus* promoted the growth of beneficial bacteria, including Prevotella, Megamonas, and Bacteroides during the in vitro fermentation process [86]. *Hirsutella sinensis* mycelium (HSM) polysaccharides showed protective effects on gut integrity by selectively promoting the growth of a commensal bacterium, Parabacteroides goldsteinii, whose level was reduced in HFD-induced mice. HSM polysaccharides reduced inflammation associated with metabolic disorders, relieved insulin resistance, and improved lipid metabolism to treat obesity and T2DM [87].

**Table 1:** Interactions between plant polysaccharides and the gut microbiota in diabetic mellitus.

Polysaccharide Source	Mw (kDa)	Monosaccharide Composition	Research Model	Anti-Diabetic Activity	Gut Microbiota Modulation	Reference
*Apocynum venetum* leaves	289.2	Man, Rha, GluA, GalA, Glu, Gal, and Ara with a ratio of 2.90:28.06:1.92:21.72:10.47:26.69:8.42;	HFD and STZ induced C57BL/6J male mice	↓ liquid intake; liver and heart indexes; insulin resistance; GSP; TG; LDL-C; NEFA; ALT; AST; ↑ liver glycogen; glucose tolerance; β-cell function; CAT, SOD; GSH; SCFAs (acetate; butyrate); relieve the histopathological injuries of liver and pancreas	↓ Firmicutes to Bacteroidetes ratio (P); Proteobacteria (P); Enterococcus (G); Klebsiella (G); Aerocuccus (G); ↑ Odoribacter (G); Anaeroplasma (G); Muribaculum (G); Parasutterella (G);	[10]
*Astragalus membranaceus*	161.15	Ara, Gal, Glu, Xyl, Man, GalA, and GluA with a ratio of 13.60:7.20:63.73:0.25:0.13:14.73:0.37	HFD and STZ induced C57BL/6J male mice	↓ body weight loss; food and water intake; FBG; GSP; FINs; TC; TG; LDL-C; LPS; TNF-α; IL-6; MDA; ALT; AST; hepatic lipid accumulation and steatosis; epididymal adipose; DAO; D-LA; ↑ glucose tolerance; HDL-C; IL-10; CAT; SOD; GSH; hepatic glycogen; relieve the histopathological injuries of pancreas and colon	↓ Helicobacter (G); Cupriavidus (G); Halomonas (G); Bacteroides (G); Odoribacter (G); Erysipelotrichaceae_Clostridium (G); Enterococcus (G); Shigella (G); Akkermansia (G); Anaeroplasma (G); AF12 (G); [Prevotella] (G); Streptococcus (G); ↑ Allobaculum (G); Lactobacillus (G);	[88]
*Berberis dasystachya*	102	Man, Ara, Glu, Gal, Xyl, and Fru with a ratio of 113.59:89.07:69.46:59.55:7.48:2.33	HFD and STZ induced Sprague Dawley male rats	↓ food and water intake; weight loss; organ index (pancreas, liver, kidneys, and heart); FBG; insulin resistance; GSP; HbAlc; MDA; NO; NOS; ↑ glucose tolerance; insulin sensitivity index; GSH-Px; SOD; SCFAs (acetic, propionic, butyric, isobutyric, valeric, and isovaleric acids); relieve the histopathological injuries of pancreas, colon tissues	↓ Bacteroidetes (P); Klebsiella (G); Ruminococcus torques group (G); Skermanella (G); Odoribacter (G); ↑ Firmicutes (P); Lactobacillus (G); Ruminococcaceae UCG-005 (G); Prevotellaceae NK3B31 group (G); Blautia (G); Ruminococcaceae NK4A214 group (G); Ruminococcus 2 (G); Eubacterium coprostanoligenes_group (G); Romboutsia (G);	[89]
*Brasenia schreberi*	50–100	-	HFD and STZ induced C57BL/6 male mice	↓ FBG; insulin resistance; TC; LDL-C; ↑ glycogen level; regulate PI3K/Akt signal pathway	↓ Firmicutes to Bacteroidetes ratio (P); Romboutsia; Desulfovibrio; ↑ Allopravotella; Lactobacillus (G); Bacteroides (G)	[90]
*Camellia sinensis*	289.734	Rha, Rib, Ara, Man, Glu, and Gal with a ratio of 1.26:3.18:4.08:1.00:1.52:3.92	HFD and STZ induced male Wistar male rats	↓ FBG; insulin resistance; TC; TG; LDL-C; FFA; Bax protein; colonic pH value; ↑ glucose tolerance; ADP; GLP-1; HDL-C; Bcl-2 protein; SCFAs (acetic acid; propionic acid; n-butyric acid; i-butyric acid and n-valeric acid); relieve the histopathological injury of pancreas	↓ Bacteroidetes (P); ↑ Proteobacteria (P); Fluviicola (G); Roseburia (G); Victivallis (G); Lachnospira (G);	[91]
Coix seed	13.285	Fuc, Rha, Ara, Gal, Glu, Xyl, Man, Fru, Rib, GalA, GulA, GluA, and ManA with a ratio of 0.25:1.05:2.79:3.86:79.64:2.75:3.54:0.31:0.08:4.26:0.31:0.81:0.18	HFD and STZ induced C57BL/6J male mice	↓ FBG; body weight loss; food intake; insulin resistance; TC; TG; LDL-C; ↑ glucose tolerance; HDL-C; SCFAs; ZO-1 expression; relieve the histopathological injury of colon; regulate IGF1/PI3K/AKT signaling pathway	↓ Firmicutes (P); Helicobacter; ↑ Bacteroidetes (P); Lactobacillus (G), Akkermansia (G), Bacteroides (G); Bifidobacterium (G);	[92]
*Lycium barbarum*	98.0	Rha, Ara, Xyl, Man, Glu, Gal, GluA, and GalA with a ratio of 0.23:1.90:0.26:0.20:1.0:1.26:0.44:1.49	HFD and STZ induced C57BL/6 male mice	↓ body weight loss; food and water intake; FBG; insulin resistance; HbA1c; GSP; insulin; TC; TG; LDL-C; ALT; AST; MDA; IL-6; IL-1b; TNF-α; LPS; ↑ glucose tolerance; insulin sensitivity; GLP-1; PYY; TBA; HDL-C; CAT; SOD; GSH-Px; TAOC; β cell function; glycogen; SCFAs (acetate, propionate, butyrate, isobutyrate, valerate, iso-valerate, and isovalerate); relieve the histopathological injuries of pancreas, liver, and skeletal muscle	↓ Firmicutes (P); Allobaculum (G); Dubosiella (G); Romboutsia (G); ↑ Bacteroidetes (P); Bacteroides (G); Ruminococcaceae_UCG-014 (G); Mucispirillum (G); Intestinimonas (G); Ruminococcaceae_UCG-009 (G);	[93]
*Cyclocarya* *paliuru*	2.584	Glu, Ara, Gal, Man, Xyl, Rha, GalA, GluA, Fuc, and Rib in a ratio of 27.90:9.68:7.67:1.93:1.67:1.26:0.72:0.66:0.17:0.16	HFD and STZ induced Sprague-Dawley male rats	↓ FBG; insulin resistance; TC; TG; LDL-C; ↑ glucose intolerance; HDL-C; GLP-1; PYY; CAZyme subtypes; SCFAs (malonic acid, propionic acid, isobutyric acid; glutaric acid); SCFAs derivates (D-3-hydroxybutyricacid; D (-)-beta-hydroxy butyric acid and 3-hydroxycapric acid)	↓ Spirochaetes (P); Proteobacteria (P); Enterococcus_faecium (S) ↑ Firmicutes(P); Ruminococcaceae (F); Eubacteriaceae (F); Lachnospiraceae (F); Ruminococcus_bromii (S); Anaerotruncus_colihominis (S); Clostridium_methylpentosum (S); Roseburia_intestinalis (S); Roseburia_hominis (S); Clostridium_asparagiforme (S); Pseudoflavonifractor_capillosus (S); Intestinimonas_butyriciproducens (S); Intestinimonas_sp._GD2 (S); Oscillibacter_valericigenes (S); Oscillibacter_ruminantium (S)	[22]
*Nigella sativa* seed	-	-	HFD and STZ induced Kunming male mice	↓ FBG; GSP; body weight loss; TC; TG; LDL-C; MDA; IL-6; TNF-α; IL-1β; ↑ insulin; HDL-C; T-AOC; SOD; CAT; p-AKT; GLUT4; SCFAs (↑propionic acid; ↓acetic acid); relieve the histopathological injuries of liver and pancreas	↓ Firmicutes (P); Lachnospiraceae_NK4A136_group (G); f_Lachnospiraceae_Unclassified (G); ↑ Bacteroidetes (P); Bacteroides (G); f_Muribaculaceae_Unclassified (G); Lactobacillus (G);	[94]
*Moutan Cortex*	164	Glu and Ara with a ratio of 3.31:2.25	high-fat and high-sugar diet, and STZ induced SD male rats	↓ HbA1c; insulin resistance; renal function index (UP/24 h, Scr, BUN, UACR); IL-6; isovaleric acid; ↑ GLP-1; expression of tight junction proteins (ZO-1, Claudin-1, Occludin); IL-10; SCFAs (acetic acid, propionic acid, butyric acid); relieve the histopathological injuries of kidney, ileum, colon	↑ Verrucomicrobia (P); Mollicutes (G), Bacteroidia (G); Lactobacillus (G); Akkermansia (G); Ruminococcaceae_UCG-014 (G); Muribaculaceae_unclassified (G)	[95]
*Setaria italica*	-	Man, Rha, Gal, Xyl, and Ara in a ratio of 0.72:0.59:76.26:1.03:0.83	HFD and STZ induced Kunming male rats	↓ body weight loss; FBG; TC; TG; LDL-C; MDA ↑ glucose tolerance; HDL-C; CAT; SOD; GSH-Px; SCFAs (acetic acid, propionic acid, butyric acid); relieve the histopathological injuries of liver and pancreas	↓ Firmicutes (P); Verrucomicrobiota (P); Peptostreptococcales-Tissierellales (O); Lachnospirales (O); Romboutsia (O); Bacteroides (O); ↑ Proteobacteria (P); Pseudomonadales (O); Pseudomonas (G); Alloprevotella (G); Akkermansia (G); Alistipes (G)	[11]

P: phylum; O: order; F: family; G: genus; S: species.

**Table 2:** Interactions between fungi polysaccharides and gut microbiota in diabetic mellitus.

Polysaccharide Source	Mw (kDa)	Monosaccharide Composition	Research Model	Anti-Diabetic Activity	Gut Microbiota Modulation	Reference
*Auricularia auricula-judae*	-	Man, Glu, Gal, Rha, Xyl, and Fru in a ratio of 62:12.6:4:1.31:4: 3.8	HFD and STZ induced C57BL/6 male mice	↓ relative epididymal fat weight; FBG; insulin resistance; TC; TG; LDL-C; lipid accumulation; ALT; AST; TNF-α; IL-6; ↑ glucose tolerance; GLP-1; HDL-C; relieve the histopathological injuries of liver and pancreas; regulate the AKT/AMPK signaling pathways; enrich KEGG pathways	↓ Firmicutes to Bacteroidetes ratio (P); Proteobacteria (P); Alistipes; Allobaculum; unidentified_Lachnospiraceae; Clostridium; ↑ Lactobacillus; Oscillospira; Rikenella; Bacteroides; Lactococcus; Odoribacter; Ruminococcus; Anaerotruncus;	[96]
*Ganoderma lucidum*	11.079	Ara, Gal, Glu, Xyl, Man, Rib, and Rha in a ratio of 5.32:5.47:57.63:0.84:25.41:1.95:3.38	HFD and STZ induced Kunming male mice	↓ body weight loss; liver and kidney weight; FBG; insulin resistance; LDL-C; TC; TG; ALT; AST; MDA; fat accumulation; ↑ HDL-C; GSH-Px; SOD; liver glycogen; relieve the histopathological injuries of liver and pancreas	↓ Firmicutes (P); Proteobacteria (P); Desulfovibrionaceae (F); Bacteroidaceae (F); Lachnospiraceae (F); Lactobacillaceae (F); _f__Desulfovibrionaceae (G); Acetatifactor (G); Lactobacillus (G); ↑ Bacteroidetes (P); Epsilonbacteraeota (P); Muribaculaceae (F); Helicobacteraceae (F); Peptococcaceae (F); Lactobacillaceae (F); Ruminococcaceae (F); Prevotellaceae(F); Alloprevotella (G); Ruminiclostridium_5 (G); f__Peptococcaceae (G); Tyzzerella (G);	[97]
*Cordyceps militaris*	87.8	Man, Gal, and Glu in a ratio of 2.2:15.1:1	HFD and STZ induced C57BL/6 male mice	↓ food and water intake; FBG; insulin resistance; LEP; TC; TG; ALT; AST; BUN; Cr; LPS; TNF-α; IL-1β; IL-6; ↑ glucose tolerance; GLP-1; ADP; colon tight junction proteins (Claudin1, Occludin, and ZO-1); relieve the histopathological injuries of liver, kidney, pancreas and colon; inhibit TLR4/NF-κB pathway	↓ Firmicutes/Bacteroidetes ratio (P); Verrucomicrobiota (P); Proteobacteria (P); Desulfobacterota (F); Escherichia-Shigella (G); Enterococcus (G); ↑ Bacteroidota (P); Campilobacterota (F); Actinobacteriota (F); norank_f_Muribaculaceae (G); Lachnospiraceae_NK4A136_group (G); norank_o__Clostridia_UCG-014 (G); Alistipes (G), Helicobacter (G); Eubacterium_xylanophilum_group (G)	[98]
*Grifola frondosa*	12,600	Ara, Man and Glu in a ratio of 3.79:1.00:49.70.	high-fat, high-sugar diet and STZ induced ICR male mice	↓ FBG; HbA1c; expression of JNK1/2; ↑ glucose tolerance; β-cells function; expression of IRS1and PI3K; GLUT4; relieve the histopathological injuries of liver and kidney	↓ Firmicutes (P); Proteobacteria (P); ↑ Bacteroidetes (P); Porphyromonas gingivalis (S); Akkermansia muciniphila (S); Lactobacillus acidophilus (S); Tannerella forsythia (S); Bacteroides acidifaciens (S); Roseburia intestinalis (S)	[99]
*Morchella esculenta*	-	Man, Rib, Rha, GluA, GalA, Glu, Gal, Ara, and Fuc in a ratio of 5.77:0.263:0.018:0.036:0.006:81.35:3.543:8.99:0.016	HFD and STZ induced BALB/c male mice	↓ body weight loss; FBG; insulin resistance; IL-6; IL-1β; TNF-α; LPS; ↑ glucose tolerance; colon tight junction proteins (ZO-1, occludin, and claudin-1); MUC2 protein; relieve the histopathological injuries of colon; regulate the KEGG pathways	↓ Firmicutes (P); Corynebacterium (G); Facklamia (G); Corynebacteriaceae (F); Actinomyceletes (C); Staphylococcaceae (S); ↑ Actinobacteria (P); Lactobacillus (G); Lactobacillaceae (F); Lachnospiraceae (F); Enterobacteriaceae (F); Lactobacilliaceae (S)	[100]

P: phylum; F: family; G: genus; S: species.

In a recent study, we discovered that soluble dietary fiber from *Lentinula edodes* byproducts combined with Lactobacillus plantarum LP90 repaired intestinal epithelial injury in dextran sulfate sodium-induced colitis mice. Additionally, the synbiotic therapy enhanced the production of butyric acid and upregulated the expression of tight junction proteins [101].

### 2.3. Polysaccharides Extracted from Marine Organisms

Marine organisms are a huge source of material for drug development. Many marine organisms are used as food ingredients and supplements due to their great taste and nutrition. Researchers are paying more attention to marine active ingredients because of the unique environmental characteristics like the high salt, high pressure and the lack of light. For example, *Ishige okamurae* extract showed multiple activities such as anti-obesity, anti-diabetic, anti-inflammatory and antioxidant activities in vitro or in mice [102,103,104,105]. As one of the major components of the active ingredients from marine organisms, polysaccharides play various activities. Two polysaccharides (laminaran SdL, fucoidan SdF) extracted from brown alga *Sargassum duplicatum* had different structures and fucoidan SdF showed anticancer effect in vitro on colon cancer cells [106]. *Undaria pinnatifida* polysaccharides exhibited α-glucosidase inhibitory activity in vitro and alleviated HFD/STZ-induced hyperglycemia by increasing glucose tolerance, relieving insulin resistance and the histopathological injuries of pancreas and liver [107]. They also showed anti-inflammatory activity by inhibiting IFN-γ expression in mice [108]. In addition to the examples above, the sources, structures, anti-diabetic effects and gut microbiota regulation function of polysaccharides from marine organisms are shown in Table 3.

An in vitro digestion experiment showed that polysaccharides from *Gracilaria lemaneiformis* were mostly degraded in the fermentation process, promoting the production of SCFAs and inhibiting the growth of the Firmicutes community [109]. Another in vitro experiment illustrated that fermentation of fucosylated chondroitin sulfate from *Stichopus chloronotus* contributed to Bacteroidetes and Fusobacteria and benefited host health by lowering the F/B ratio in turn [110].

Experiments in vivo showed similar results. For instance, the seaweed polysaccharides laminaran and ulvan were slowly fermented by Bifidobacterium as well as stimulated the growth of Bifidobacterium and promoted the production of acetate, propionate and lactate [111]. The mixture of algal polysaccharides ulvan and astaxanthin increased the level of Bacteroidia, Bacilli, Clostridia, and Verrucomicrobia [112]. Sulfated sea cucumber *Stichopus japonicus* significantly enriched the relative abundance of Parabacteroides and Akkermansia, as well as reduced the level of Proteobacteria [113,114]. *Sargassum fusiforme* polysaccharides decreased the relative abundance of Bacteroidetes and increased that of Oscillospira, Mucispirillum, and Clostridiales [115]. *Laminaria japonica* polysaccharides upregulated Turicibacter, which produced SCFAs, especially lactic acid [116].

**Table 3:** Interactions between marine polysaccharides and gut microbiota in diabetic mellitus.

Polysaccharide Source	Mw (kDa)	Monosaccharide Composition	Research Model	Anti-Diabetic Activity	Gut Microbiota Modulation	Reference
*Dictyopteris divaricata*	63.06	Man, Rib, Rha, GluA, Glu, Gal, Xyl, Ara, and Fuc in a ratio of 15.02:9.90:1.28:17.54:1.86:17.19:4.54:0.55	high sugar diet and STZ induced Balb/c male mice	↓ body weight loss; food and water intake; FBG; PBG-2h; insulin resistance; TC; TG; LDL-C; IL-1β; IL-2; IL-6, TNF-α; IFN-γ; MDA; ↑ glucose tolerance; β cell function; HDL-C; SOD; MUC-2; ZO-1; tight junction proteins (Occludin; Claudin-1); IRS-1; relieve histopathological injury of colon	↓ Bacteroidetes (P); Proteobacteria (P); Actinobacteria (P); S24-7 (F); Paraprevotellaceae (F); Odoribacteraceae (F); Corynebacteriaceae (F); Bacteroides (G); Corynebacterium (G); Ruminococcus (G); Parabacteroides (G); ↑ Firmicutes (P); Lactobacillus (G); Prevotella (G); Oscillospira (G); Lactobacillaceae (F); Ruminococaceae (F); Lachnospiraceae (F); Rikenellaceae (F)	[117]
*Holothuria* *leucospilota*	52.8	Rha, Fuc, Glua, galactose, Glu, and Xyl in a ratio of 39.1:35.7:10.7:8.4:4.2:1.8	GK male rats and age-matched Wistar rats	↓ FBG; TC; TG; LDL-C; insulin; LEP; CD36; Bax; ↑ glucose tolerance; HDL-C; adiponectin; GLP-1; PI3K; AKT; PPAR-α; GLUT4; Bcl-2; SCFAs (acetic, butyric acid, pentanoic acid); relieve histopathological injuries of pancreas, colon	↓ Firmicutes (P); Proteobacteria (P); Spirochaetes (P); Actinobacteria (P); Bilophila (G); Bifidobacterium (G); Mucispirillum (G); Colinsella (G); Gemella (G); Treponema (G); Anaerobiospirillum (G); Aggregatibacter (G); Facklamia (G); Lactobacillus (G); ↑ Bacteroidetes (P); TM7 (P); Cyanobacteria (P); Tenericutes (P); Ruminococcus (G); Holdemania (G); Clostridium (G); Helicobacter (G); Turicibacter (G); Paraprevotella (G); Bacteroides (G); Faecalibacterium (G)	[118]
*Ulva lactuca*	224	Rha, GluA, Gal, and Xyl in a ratio of 32.75:22.83:1.07:6.46	high-fat high sugar diet and STZ induced ICR male mice	↓ FBG; body weight loss; MDA; ↑ glucose tolerance; CAT; SOD; GSH-PX; relieve the histopathological injury of liver; regulate JAK/STAT3 pathway	↓ Firmicutes (P); ↑ Bacteroidetes (P); Actinobacteria (P); s_weissella_cibaria (G); g_Candidatus_Saccharimonas (G); f_Saccharimonadaceae (G); c_Saccharimonadia (G); o_Saccharimonadales (G)	[119]
*Macrocystis pyrifera*	342.1	Gal, Fuc, Man, and GluA in ratio of 29.29:27.59:21.24: 16.99	high-fat, high-sugar and STZ induced Sprague Dawley male rats	↓ body weight loss; glucose; HbA1c; insulin resistance; TG; TC; LDL-C; AST; ALT; BUN; Cr; TNF-α; IL-6; MDA ↑ glucose tolerance; GSH-Px;	↓ Escherichia–Shigella (G); ↑ Muribaculaceae_norank (G); Akkermansia (G); Bifidobacterium (G); Lactobacillus (G); Olsenella (G); Lachnospiraceae_NK4A136_group (G); Ruminococcaceae_UCG-014 (G); Ruminococcus_1 (G); Eubacterium_coprostanoligenes_group (G)	[120]
*Onchidium struma*	8–14	Ara, Man and Glu in a ratio of 3.79:1.00:49.70.	high-sucrose high-fat diet and STZ induced Kungming male mice	↓ body weight loss; FBG; blood glucose; FIN level; HOMA-IRI; TC; TG; LDL-C; GSP; IL-6; LPS; TNF-α; GSK-3β; ↑ daily intake; glucose tolerance; FER value; HOMA-ISI; HOMA-β; HDL-C; IL-10; mRNA expression (PI3K, AKT-1, mTOR, GLUT-2); SCFAs (acetate, propionate, isobutyrate, butyrate, isovalerate, valerate); relieve the histopathological injury of liver	↓ Firmicutes to Bacteroidetes (P); Lachnoclostridium; Parabacteroides; ↑ Alipipes; Lactobacillus	[121]

P: phylum; F: family; G: genus.

## 3. Mechanism of the Anti-Diabetic Effects of Polysaccharides through Regulating Gut Microbiota

**Figure 2:**
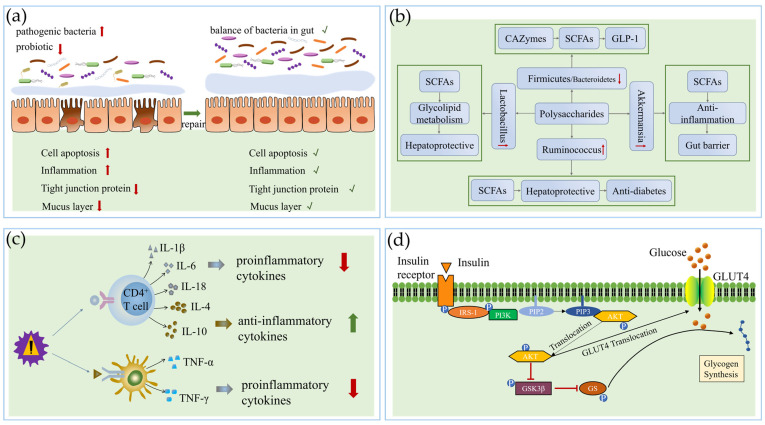
Mechanisms of polysaccharides: (**a**) repairing gut barrier, (**b**) changing gut microbiota composition and metabolites, (**c**) regulating anti-inflammatory activity and immune function, and (**d**) regulating signal pathways. The up-pointing or right-pointing arrow (red or green) means increase, and the down-pointing arrow (red) means decrease. √ means improvement.

The gut microbiota is closely related to human health maintenance. Research has shown that there is an important relationship between the gut microbiota and DM [122]. The gut microbiota forms a network regulating the gut barrier, the production and utilization of metabolites, and the immune function of the gut [123]. It also influences the use of glucose and the conversion of glycogen, and its composition is related to the progression of insulin resistance in T2DM as well as the development of complications of DM [124,125]. Below, the anti-diabetic mechanisms of polysaccharides through regulating the gut microbiota are summarized and are shown in Figure 2.

### 3.1. Repairing the Gut Barrier

The mucus layer and the intestinal apical junctional protein complex make up the complex gut barrier, which protects host health from many acute and chronic illnesses [126]. Inflammatory bowel disease (IBD), colon cancer, and many other illnesses were related to damage to the gut barrier [127]. In diabetic mice, the destruction of the gut barrier was shown in a reduction in the number of goblet cells, shorter and irregular villi, a reduction in mucosal space, an influx of inflammatory cells, and weakened epithelial cells [121,128,129]. When the gut barrier was damaged, its function was decreased or even disturbed. Many studies have shown that polysaccharides showed great protective effects on gut barrier integrity. For example, *Pleurotus eryngii* polysaccharide interacted with intestinal mucus layer after in vitro fermentation by the gut microbiota [130]. *Ganoderma atrum* polysaccharides maintained the intestinal barrier and its permeability in rats [131]. The mixture of hawthorn flavonoids and two kinds of polysaccharides from *Auricularia auricula* and *Tremella* improved injuries to the intestinal barrier and epithelial cells in rats [132]. Dandelion polysaccharides repaired the intestinal barrier and improved the structure of the gut microbiota in mice [133].

Polysaccharides can also relieve the injury of the gut barrier through improving tight junction proteins. Cultured *Cordyceps sinensis* polysaccharides, *Dictyopteris divaricate* polysaccharides, and *Phellinus linteus* polysaccharides upregulated tight junction proteins such as occludin, claudin-1, and ZO-1 in intestinal barrier injury in mice, maintaining the gut structure and barrier permeability in mice or rats [47,121,134]. Mucin-2 protein (MUC2) is a kind of heavily glycosylated mucin proteins secreted by intestinal cells protecting the human gut from potentially harmful bacteria and substances [135]. Polysaccharides from the fruit of *Lycium barbarum* increased the mRNA expression of MUC2 in mouse colon [136]. Quinoa seed polysaccharides increased the production of intestinal mucus in rats, protecting host from infection [137].

### 3.2. Changing Gut Microbiota Composition and Metabolites

Research found that the production of bacteria-derived metabolite damaging to human health was strongly associated with gut dysbiosis reversal [89]. Polysaccharides, regarded as a kind of prebiotics, were found to promote the growth and/or activity of beneficial bacteria and to suppress pathogenic bacteria in rat gut [47]. Polysaccharides also influenced the metabolism of SCFAs and other metabolites indirectly by changing the composition and diversity of the gut microbiota in rats [138]. The metabolites of the gut microbiota influenced the gut and system function by circulation or acting as ligands for cell receptors [139]. For example, increased expression of GPCR 41/43 in the intestinal L cells caused by higher SCFAs may cause the production of GLP-1 [140].

At the phylum level, the Gram-positive Firmicutes and Gram-negative Bacteroidetes were two dominant bacteria in the gut microbiota, comprising more than 90% of total 16S rRNA-targeted sequences from bacteria [141]. Firmicutes and Bacteroidetes had many carbohydrate metabolism pathways, which promoted the expression of CAZymes to degrade most polysaccharides to produce SCFAs, participating in the regulation of glycol metabolism [142,143]. One study found that the F/B ratio was related to metabolic disorders, and compared with a normal group, the F/B ratio was considerably increased in diabetic model mice [68]. Regulation of the F/B ratio is one important way for polysaccharides to modulate the composition of the gut microbiota and improve the gut metabolites.

*Dendrobium officinale* leaf polysaccharides change gut microbiota composition and derived microbial compounds. They decrease the F/B ratio and upregulate butyrate production to repair the intestinal microenvironment in mice [128]. The treatment of polysaccharide from *Cyclocarya paliurus* leaves attenuated the decrease in the F/B ratio induced by diabetes, while in nondiabetic rats, polysaccharide administration did mot have the same effect [144]. *Astragalus membranaceus* polysaccharide treatment decreased the F/B ratio and promoted the production of acetic acid, butyric acid, and propanoic acid in feces from db/db mice. Konjac glucomannans (KGMs) increased Bacteroidetes and decreased Firmicutes abundance in diabetic mice. KGMs increased the abundance of Muribaculaceae, part of Bacteroidetes, and produced SCFAs to improve pancreatic β-cell function and reduce the release of proinflammatory cytokines [145].

It was also found that Akkermansia is closely related to human health and some DM related indices and is a potential probiotic for the treatment of diabetes [146]. Akkermansia is a kind of representative bacterium in the mucus layer of the intestine that participates in the degradation process of mucin, thereby protecting the intestinal mucosal barrier and reducing protein deposition [147]. Metformin therapy enhances the relative abundance of Akkermansia in mice [148]. Polysaccharides from pumpkin, gougunao tea, and *Gastrodia elata* increased Akkermansia in T2DM mice [149,150,151].

Ruminococcus, which is abundant and common in the mammalian gut environment, can degrade polysaccharides to produce SCFAs and is a predominant acetogen producing acetic acid and propionate [152]. It was reported that Ruminococcaceae UCG-005 is a key genus for protecting against diabetes [144]. One study found that Ruminococcus was negatively connected with FBG and positively related to body weight [74]. Ruminococcaceae UCG-014 and UCG-005 genera were negatively correlated with indices including liver weight, AST and ALT levels, hepatic steatosis and inflammation degree, which revealed their hepatoprotective effect [153]. *Grifola frondosa*, *Ganoderma atrum* and *Cordyceps militaris* polysaccharide supplementation enriched the relative abundance of Ruminococcus in HFD rats [154].

Furthermore, a number of studies have shown that reducing the amount of Alistipes in the gut may decrease the generation of antimicrobial peptides, which in turn promotes the colonization of harmful microbes on the gut barrier and results in liver illnesses. For example, Alistipes play a crucial part in the prevention of dextran sulfate sodium-induced colitis in mice [155]. Polysaccharides from *Grifola frondose* and *Lentinula edodes* significantly increased the abundance of Alistipes in mice [156,157]. *Auricularia auricula* polysaccharides markedly increased the level of Bifidobacterium animalis, Morchella esculenta, and *Inonotus obliquus* polysaccharides enhanced the level of Lactobacillus in mice [77,158,159].

SCFAs belong to beneficial metabolites, positively correlated with the health of the body. *Phellinus linteus* polysaccharides increased the abundance of Roseburia, Lachnospiraceae-NK4A136, Lachnospiraceae-UCG-006, and Prevotella9 that decomposed fiber polysaccharides and produced SCFAs in STZ-induced male Sprague Dawley rats [134]. Similarly, *Ganoderma lucidum* and *Poria cocos* polysaccharides increased SCFA-producing bacteria like Prevotella and Paraprevotella clara, and *Poria cocos* polysaccharides greatly increased Bacteroides xylanolyticus, a xylan-degrading bacterium in mice [160].

### 3.3. Regulating Anti-Inflammatory Activity and Immune Function

Inflammation and disorder of the immune system in the host, e.g., IBD, systemic lupus erythematosus, asthma, arthritis, and many other diseases, are impacted by changes in the normal gut microbiota [161,162]. A low-grade inflammatory and autoimmune condition that is closely linked to T2DM may be caused directly by changes in the gut microbiota’s composition. According to the results of Pearson analysis, the relative abundance of some of the gut microbiota was closely correlated with indices of immunological organs like the spleen and thymus and immunoglobulins like IgG and IgM, and CD4^+^ T cells [163]. Investigation of the connection between the gut microbiota and biochemical profiles showed Bifidobacterium was involved in inflammation and Fusobacterium modulated host immune responses [93,164]. Similarly, relevant studies showed that members of the Lachnospira family in the human gut express two “superantigens” that stimulate the IgA response and are crucial for intestinal homeostasis [165]. Another study found that an increase in Proteobacteria resulted in immune dysregulation in the host [166]. *Agaricus blazei* Murill polysaccharides decreased Proteobacteria and increased Lachnospiraceae and Lactobacillaceae, suppressing the inflammation response in mice [167].

The intervention of polysaccharides can improve the inflammatory and immune response. For example, water extract of *Berberis kansuensis* reduced inflammatory factors such as TNF-α, IL-1β and IL-6 in diabetic mice [168]. *Lycium berry* polysaccharides downregulated proinflammatory cytokines including IL-1β and IL-18 and M1 macrophage markers and increased anti-inflammatory cytokines like IL-4 and IL-10 and M2 macrophage markers to facilitate the shifting of the epithelial immunity from the pro- to the anti-inflammatory microenvironment in mice [169]. *Nigella sativa* seed polysaccharides decreased the levels of cytokines, including IL-6, IL-1β and TNF-α [94]. *Dictyopteris divaricate* polysaccharides downregulated the expression of IL-1β, IL-2, IL-6, TNF-α and IFN-γ in T1DM mice [121].

### 3.4. Regulating the Signal Pathway

Many signal pathways have been found to be related to glucose metabolism as well as DM. For example, the mTOR signal pathway regulates energy intake and is related to metabolic disorders like T2DM [170]. The PI3K/Akt pathway is a key signaling pathway and regulates insulin signal and glucose metabolism [171,172]. Inactivation of the PI3K/Akt signaling pathway in liver reduces the synthesis of liver glycogen, resulting in insulin resistance, a typical symptom of T2DM [173]. The phosphorylation of ISR1, PI3K and Akt in succession activates GLUT4/GLUT2, leading to glucose uptake of cells [174]. Many polysaccharides relieve the symptoms of DM by regulating the PI3K/Akt signal pathway. For examples, azuki bean (*Vigna angularis*) polysaccharides control glucose metabolism and oxidative stress by considerably upregulating the expression of INSR, IRS-1, PI3K, Akt, and GLUT2 in this signal pathway [175]. *Brasenia schreberi* polysaccharides increase the expression of PI3K and Akt in T2DM mice [90]. Oligosaccharides from seaweed *Sargassum confusum* significantly upregulate the expression of IRS1 and PI3K genes and downregulate the expression of JNKs,h contributing to DM in high-fat/high-sucrose-fed hamsters [176].

Besides the signal pathways in the liver and muscle for glucose uptake, the signal pathways in the gut and other organs are also affected. Coix seed polysaccharides increase the expression of insulin-like growth factor 1 (IGF1) and insulin-like growth factor 1 receptor (IGF1R), which participates in the secretion of insulin [92]. Dietary inulin and *Lycium barbarum* polysaccharides improve the expression of Toll-like receptor 2 (TLR2) on the surface of γδ T cells, which can be recognized by the metabolites of the gut microbiota and increase the integrity of the gut microbiota in rats [177]. At the same time, a Scutellaria–Coptis herb couple downregulated the expression of Toll-like receptor 4 (TLR4) and MyD88 protein in the colon, decreasing the secretion of proinflammatory factors in mice [178]. It has been reported that polysaccharides can interact with enteroendocrine L cells directly and enhance the production of glucagon-like peptide-1 (GLP-1) through the cAMP signaling pathway and Ca^2+^/calmodulin/calmodulin-dependent protein kinase 2 signaling pathway [136]. *Cyclocarya paliuru* polysaccharides can increase the mRNA expression of G-protein-coupled receptors (GPRs) like GPR41, GPR43 and GPR109a, which are receptors of SCFAs in colon tissue in T2DM mice [118].

### 3.5. Action on Related Tissue and Organs

**Figure 3:**
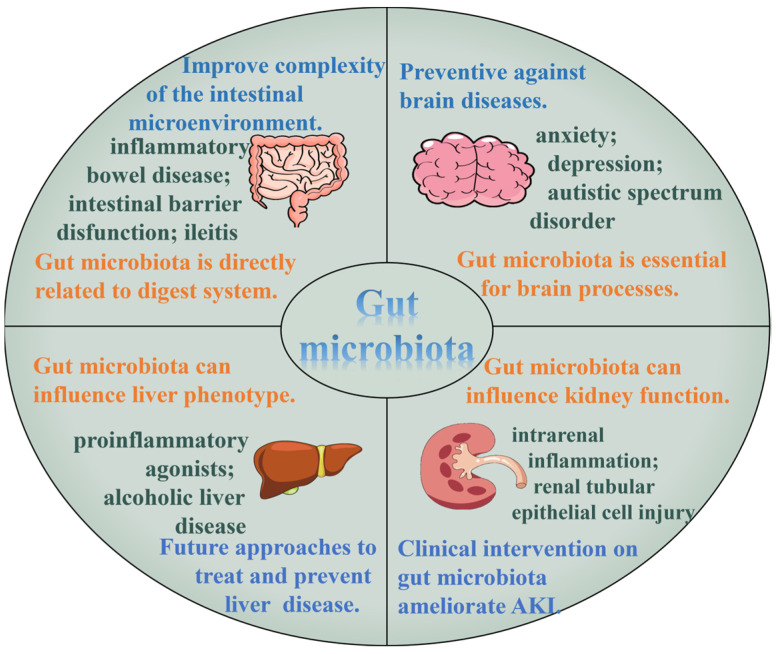
Gut microbiota and related organs.

The gut microbiota is closely related to other tissue and organs, and the mostly studied include muscle, liver, kidney and pancreas. The relationship of the gut microbiota and to these organs is shown in Figure 3.

The metabolites of the gut microbiota enter the blood and interact with receptors on muscle, liver, pancreas, and other organs, influencing their functions [179]. The microbiota–liver axis, microbiota–brain axis, microbiota–kidney, microbiota–lung, and other microbiota–organ axes have been studies [180,181,182,183,184]. For example, one study found that hepatic markers were associated with Ruminococcaceae family [153]. The regulation effects and communication with other tissue and organs of the gut microbiota has aroused researchers’ interest. Polysaccharides can play protective roles by regulating intestinal flora. For instance, water extracts of *Plukenetia volubilis* leaves restored pancreas injury, including the improvement in injured pancreas islets and pancreatic β-cells in T1DM mice [185]. Polysaccharides from red kidney bean also showed pancreas-protective effects in T2DM mice [186]. Mulberry fruit polysaccharides healed damage to the liver, kidneys and pancreas of obese diabetic mice [187].

## 4. Structure–Activity Relationship of the Anti-Diabetic Effects of Polysaccharides through Regulating Gut Microbiota

As a kind of biological macromolecule, polysaccharides have complex structures. The differences in the structure of polysaccharides, such as monosaccharide composition, molecular weight, types of glycosidic linkages and other intrinsic physicochemical characteristics, can profoundly influence their anti-diabetic effects [188]. Their structural characteristics are closely related to the fermentability of polysaccharides by the gut microbiota [189]. The structure–activity relationship of the anti-diabetic effects of polysaccharides through regulating the gut microbiota is summarized in the following section.

### 4.1. Monosaccharide Composition

Monosaccharide composition is an essential factor influencing the anti-diabetic effects of polysaccharides. In general, the hypoglycemic activity of polysaccharides increases with the complexity of monosaccharide composition [190]. For example, polysaccharides from four legume species—mung bean, azuki bean, pea and cowpea—with different monosaccharide composition show different hypoglycemic activity. Azuki bean polysaccharides with more complex monosaccharide types, including arabinose, galactose, glucose, xylose, mannose, and galacturonic acid (unique monosaccharide among the four polysaccharides), exhibit the best hypoglycemic effects in STZ-induced diabetic mice. What is more, polysaccharides from mung bean and cowpea with the same monosaccharide type but in different proportions had different hypoglycemic activity, indicating that galactose and glucose might be two important monosaccharides in the anti-diabetic process [175]. Similarly, polysaccharides from four *Gastrodia elata* plants with the same monosaccharide type but in different proportions showed different hypoglycemic activity. *G. elata Bl. f. elata* polysaccharides possessed the highest hypoglycemic activity with the lowest glucose content and highest xylose content, which indicated that xylose was also an important kind of monosaccharide participating in the anti-diabetic process [191].

### 4.2. Molecular Weight

Molecular weight significantly influences the anti-diabetic and gut microbiota regulation activity of polysaccharides [192]. Typically, polysaccharides have stronger anti-diabetic activity when their molecular weight is lower [76]. For example, blackberry polysaccharides with lower molecular weight were more easily broken down and consumed by the gut microbiota and exhibited better prebiotic effects [193]. Pectin from artichoke and citrus promoted the growth of Bifidobacterium and Lactobacillus, and the enzymatically modified pectin with lower molecular weight showed higher activity [194]. Rhamnogalacturonan-I (RG-I)-enriched pectin (WRP) from citrus and its depolymerized fraction (DWRP) with lower molecular weight showed different gut microbiota regulation function. WRP increased the abundance of Ruminococcaceae, while the abundance of prebiotics like Bifidobacterium and Lactobacillus were significantly by DWRP [195].

There are also some polysaccharides with higher or medium molecular weight showing higher hypoglycemic activity. For instance, Chinese yam polysaccharides HSY-I (>50 kDa) and HSY-II (10 to 50 kDa) exhibited hypoglycemic activity by digestion resistance or β-insulin cell repair. HSY-III (<10 kDa) had no hypoglycemic effect [196]. Peach gum polysaccharides (PGPs) promoted the growth of Bacteroides and Parabacteroides. PGPs with high molecular weight played the most significant role in the production of SCFAs [197]. Konjac glucomannan with medium molecular weight displayed higher hypoglycemic effects and regulated the gut microbiota by modulating gut microbiota composition and improving gut microbiota-related metabolites [145].

*Laminaria japonica* polysaccharides with higher (HLJP) and lower (LLJP) molecular weight showed different gut microbiota and metabolite regulation function. The function of HLJP focused more on the regulation of SCFAs and metabolites, while that of LLJP focused more on the proliferation probiotic of gut microbiota like Akkermansiaceae [198].

Numerous polysaccharide characteristics, such as viscosity and advanced structure, are correlated with molecular weight. More research is needed to determine the relationship between polysaccharide molecular weight and anti-diabetic activity.

### 4.3. Types of Glycosidic Linkage

The orientation and position of glycosidic bonds affect the anti-diabetic activity of polysaccharides. For example, fucoidan from Ascophyllum nodosum with alternating (1→3) and (1→4)-α-l-fucose showed better α-glucosidase inhibitory activity than that with repeated (1→3)-α-l-fucose from Fucus vesiculosus, while the inhibitory effect on α-amylase was opposite to α-glucosidase [199]. The glycosidic linkage also influenced the production of SCFAs. The fermentation of polysaccharides with diglucose α-(1→1) structure is positively related to the production of butyrate and negatively related to the production of acetate. Fermentation of polysaccharides with diglucose β-(1→4) structure produces more butyrate, propionate and butyrate than diglucose β-(1→4) structure [200]. As for gut regulation, fucogalactan sulfate from Laminaria japonica, with more branched sugar residues like (1→2, 3, 4)-linked β-D-ManpA and sulfate ester groups, showed better effects in terms of the proliferation of beneficial bacteria and production of SCFAs and other metabolites than mannogluconic acid [201].

## 5. Conclusions and Prospects

The gut microbiota is closely related to DM and is becoming a new target in the treatment of DM. In this paper, we discussed the anti-diabetic effects of polysaccharides through regulation of the gut microbiota. Most polysaccharides cannot be digested by the human body directly, but are fermented by the gut microbiota to produce SCFAs and other metabolites. The interaction between polysaccharides and the gut microbiota can result in the formation of different metabolites and remodeling of the microbiota. The proposal of the gut–organ axis also provides a model for the understanding and treatment of human diseases. However, due to the complexity of the gut microbiota and the uncertainty of polysaccharide structure, the exact interaction between polysaccharides and the gut microbiota still needs further study. It is necessary to give a more detailed description of polysaccharide structure so that we can gain a clearer picture of how polysaccharides influence the gut microbiota and improve DM. The regulatory functions of the gut microbiota, including the metabolism process and the metabolites themselves and their interactions with other organs or tissue types, also need further study.

## Data Availability

Data sharing is applicable.

## Abbreviations

STZ (streptozotocin); IFN-γ (interferon γ); GPCR 41/43 (G-protein-coupled receptor 41/43); FBG (fasting blood glucose); GSP (glycosylated serum protein); TG (triglyceride); LDL-C (low-density-lipoprotein cholesterol); NEFA (non-esterified fatty acid); ALT (glutamic pyruvic transaminase); AST (glutamic oxaloacetic transaminase); CAT (catalase); SOD (superoxide dismutase); GSH (glutathione); FINS (fasting insulin); TC (total cholesterol); LPS (lipopolysaccharide); TNF-α (tumor necrosis factor α); IL-2/4/6/8/10/18/1β/1b (interleukin-2/4/6/8/10/18/1β/1b); MDA (malondialdehyde); DAO (diamine oxidase); D-LA (d-lactic acid); HDL-C (high-density-lipoprotein cholesterol); HbAlc (glycosylated hemoglobin, type A1C); NO (nitric oxide); NOS (nitric oxide synthase); GSH-Px (glutathione peroxidase); FFA (free fatty acid); Bax (Bcl2-associated X); ADP (adenosine diphosphate); Bcl-2 (B-cell lymphoma 2); ZO-1 (zonula occludens 1); PYY (peptide YY); TBA (total bile acid); TAOC (total antioxidant capacity); UP (urine protein); Scr (serum creatinine); BUN (blood urea nitrogen); UACR (urine microalbumin:creatinine ratio); LEP (leptin); Cr (creatinine); LPS (lipase); JNK (c-Jun N-terminal kinase); PBG (postprandial blood glucose); CD36 (cluster of differentiation 36); PPAR-α (peroxisome proliferator-activated receptor α); HOMA-IRI (HOMA insulin resistance index); FER (food efficiency rate); IgA/M (immunoglobulin A/M).

## References

[1] ElSayed N.A., Aleppo G., Aroda V.R., Bannuru R.R., Brown F.M., Bruemmer D., Collins B.S., Hilliard M.E., Isaacs D., Johnson E.L. (2023). 2. Classification and Diagnosis of Diabetes: Standards of Care in Diabetes-2023. Diabetes Care.

[2] Craciun C.I., Neag M.A., Catinean A., Mitre A.O., Rusu A., Bala C., Roman G., Buzoianu A.D., Muntean D.M., Craciun A.E. (2022). The Relationships between Gut Microbiota and Diabetes Mellitus, and Treatments for Diabetes Mellitus. Biomedicines.

[3] Giovannini P., Howes M.J.R., Edwards S.E. (2016). Medicinal plants used in the traditional management of diabetes and its sequelae in Central America: A review. J. Ethnopharmacol..

[4] Baek S.M., Kim K., Kim S., Son Y., Hong H.S., Yu S.Y. (2020). SP prevents T2DM complications by immunomodulation. Sci. Rep..

[5] Yi X.R., Dong M.S., Guo N.F., Tian J.L., Lei P., Wang S., Yang Y.F., Shi Y. (2023). Flavonoids improve type 2 diabetes mellitus and its complications: A review. Front. Nutr..

[6] Wu Z., Zeng W.Z., Zhang X., Yang J.F. (2022). Characterization of Acidic Tea Polysaccharides from Yellow Leaves of Wuyi Rock Tea and Their Hypoglycemic Activity via Intestinal Flora Regulation in Rats. Foods.

[7] Perreault L., Pan Q., Aroda V.R., Barrett-Connor E., Dabelea D., Dagogo-Jack S., Hamman R.F., Kahn S.E., Mather K.J., Knowler W.C. (2017). Exploring residual risk for diabetes and microvascular disease in the Diabetes Prevention Program Outcomes Study (DPPOS). Diabet. Med..

[8] Pagano G., Polychronis S., Wilson H., Giordano B., Ferrara N., Niccolini F., Politis M. (2018). Diabetes mellitus and Parkinson disease. Neurology.

[9] Schmidt A.M. (2019). Diabetes Mellitus and Cardiovascular Disease Emerging Therapeutic Approaches. Arter. Throm. Vas..

[10] Yuan Y., Zhou J., Zheng Y., Xu Z., Li Y., Zhou S., Zhang C. (2020). Beneficial effects of polysaccharide-rich extracts from Apocynum venetum leaves on hypoglycemic and gut microbiota in type 2 diabetic mice. Biomed. Pharmacother..

[11] Zhang J.H., Wang W.J., Guo D.Y., Bai B.Q., Bo T., Fan S.H. (2022). Antidiabetic Effect of Millet Bran Polysaccharides Partially Mediated via Changes in Gut Microbiome. Foods.

[12] Cui J.F., Ye H., Zhu Y.J., Li Y.P., Wang J.F., Wang P. (2019). Characterization and Hypoglycemic Activity of a Rhamnan-Type Sulfated Polysaccharide Derivative. Mar. Drugs.

[13] Kholiya F., Chatterjee S., Bhojani G., Sen S., Barkume M., Kasinathan N.K., Kode J., Meena R. (2020). Seaweed polysaccharide derived bioaldehyde nanocomposite: Potential application in anticancer therapeutics. Carbohydr. Polym..

[14] Roszczyk A., Turlo J., Zagozdzon R., Kaleta B. (2022). Immunomodulatory Properties of Polysaccharides from Lentinula edodes. Int. J. Mol. Sci..

[15] Wei H., Shi Y.Q., Yuan Z.X., Huang Z.N., Cai F.H., Zhu J.F., Zhang W.W., Li J., Xiong Q.P., Wang Y.P. (2021). Isolation, Identification, and Anti-Inflammatory Activity of Polysaccharides of Typha angustifolia. Biomacromolecules.

[16] Zhao Z.K., Yu H.L., Liu B., Wang H., Luo Q., Ding X.G. (2016). Antioxidative mechanism of Lycium barbarum polysaccharides promotes repair and regeneration following cavernous nerve injury. Neural Regen. Res..

[17] Fan Y.M., Zhou X.F., Huang G.L. (2022). Preparation, structure, and properties of tea polysaccharide. Chem. Biol. Drug Des..

[18] Ullah S., Khalil A.A., Shaukat F., Song Y.D. (2019). Sources, Extraction and Biomedical Properties of Polysaccharides. Foods.

[19] Alba K., Dimopoulou M., Kontogiorgos V. (2021). Baobab polysaccharides as emulsifiers. LWT.

[20] Sun B.N., Yu S., Zhao D.Y., Guo S.H., Wang X.H., Zhao K. (2018). Polysaccharides as vaccine adjuvants. Vaccine.

[21] Yang Z.J., Liu W.W., Liu H.M., Li R., Chang L., Kan S.N., Hao M., Wang D.X. (2022). The applications of polysaccharides in dentistry. Front. Bioeng. Biotech..

[22] Yao Y., Yan L.J., Chen H., Wu N., Wang W.B., Wang D.S. (2020). Cyclocarya paliurus polysaccharides alleviate type 2 diabetic symptoms by modulating gut microbiota and short-chain fatty acids. Phytomedicine.

[23] Blandino G., Inturri R., Lazzara F., Di Rosa M., Malaguarnera L. (2016). Impact of gut microbiota on diabetes mellitus. Diabetes Metab..

[24] Hermann-Bank M.L., Skovgaard K., Stockmarr A., Larsen N., Molbak L. (2013). The Gut Microbiotassay: A high-throughput qPCR approach combinable with next generation sequencing to study gut microbial diversity. BMC Genom..

[25] Tan X.J., Wang Y.Z., Gong T. (2023). The interplay between oral microbiota, gut microbiota and systematic diseases. J. Oral. Microbiol..

[26] Huttenhower C., Gevers D., Knight R., Abubucker S., Badger J.H., Chinwalla A.T., Creasy H.H., Earl A.M., FitzGerald M.G., Fulton R.S. (2012). Structure, function and diversity of the healthy human microbiome. Nature.

[27] Sonnenburg E.D., Smits S.A., Tikhonov M., Higginbottom S.K., Wingreen N.S., Sonnenburg J.L. (2016). Diet-induced extinctions in the gut microbiota compound over generations. Nature.

[28] Zhang Y.J., Li S., Gan R.Y., Zhou T., Xu D.P., Li H.B. (2015). Impacts of Gut Bacteria on Human Health and Diseases. Int. J. Mol. Sci..

[29] Fan H.X., Liu X.C., Ren Z.Y., Fei X.N., Luo J., Yang X.Y., Xue Y.Y., Zhang F.F., Liang B. (2023). Gut microbiota and cardiac arrhythmia. Front. Cell Infect. Microbiol..

[30] Shi Q.W., Dai L.L., Zhao Q., Zhang X. (2022). A review on the effect of gut microbiota on metabolic diseases. Arch. Microbiol..

[31] Masenga S.K., Kirabo A. (2023). Salt and Gut Microbiota in Heart Failure. Curr. Hypertens. Rep..

[32] Mulak A., Bonaz B. (2015). Brain-gut-microbiota axis in Parkinson’s disease. World J. Gastroenterol..

[33] AL-Ishaq R.K., Samuel S.M., Büsselberg D. (2023). The Influence of Gut Microbial Species on Diabetes Mellitus. Int. J. Mol. Sci..

[34] Bajinka O., Tan Y.R., Darboe A., Ighaede-Edwards I.G., Abdelhalim K.A. (2023). The gut microbiota pathway mechanisms of diabetes. Amb. Express.

[35] Gong P., Xiao X.Y., Wang S., Shi F.X., Liu N., Chen X.F., Yang W.J., Wang L., Chen F.X. (2021). Hypoglycemic effect of astragaloside IV via modulating gut microbiota and regulating AMPK/SIRT1 and PI3K/AKT pathway. J. Ethnopharmacol..

[36] Ma S.T., Tian S.H., Sun J., Pang X.Y., Hu Q.B., Li X.F., Lu Y.J. (2022). Broccoli microgreens have hypoglycemic effect by improving blood lipid and inflammatory factors while modulating gut microbiota in mice with type 2 diabetes. J. Food Biochem..

[37] Wang X.L., Wang X., Jiang H., Cai C., Li G.Y., Hao J.J., Yu G.L. (2018). Marine polysaccharides attenuate metabolic syndrome by fermentation products and altering gut microbiota: An overview. Carbohydr. Polym..

[38] Xie G.X., Zhong W., Zheng X.J., Li Q., Qiu Y.P., Li H.K., Chen H.Y., Zhou Z.X., Jia W. (2013). Chronic Ethanol Consumption Alters Mammalian Gastrointestinal Content Metabolites. J. Proteome Res..

[39] Sam Q.H., Ling H., Yew W.S., Tan Z.H., Ravikumar S., Chang M.W., Chai L.Y.A. (2021). The Divergent Immunomodulatory Effects of Short Chain Fatty Acids and Medium Chain Fatty Acids. Int. J. Mol. Sci..

[40] Alvarez-Mercado A.I., Plaza-Diaz J. (2022). Dietary Polysaccharides and Gut Microbiota Ecosystem. Nutrients.

[41] Riedl R.A., Atkinson S.N., Burnett C.M.L., Grobe J.L., Kirby J.R. (2017). The Gut Microbiome, Energy Homeostasis, and Implications for Hypertension. Curr. Hypertens. Rep..

[42] Li R.L., Xue Z.H., Li S.Q., Zhou J.N., Liu J.Y., Zhang M., Panichayupakaranant P., Chen H.X. (2022). Mulberry leaf polysaccharides ameliorate obesity through activation of brown adipose tissue and modulation of the gut microbiota in high-fat diet fed mice. Food Funct..

[43] Xue Z.H., Ma Q.Q., Chen Y., Lu Y.P., Wang Y.J., Jia Y.N., Zhang M., Chen H.X. (2020). Structure characterization of soluble dietary fiber fractions from mushroom Lentinula edodes (Berk.) Pegler and the effects on fermentation and human gut microbiota in vitro. Food Res. Int..

[44] Jiao R., Liu Y.X., Gao H., Xiao J., So K.F. (2016). The Anti-Oxidant and Antitumor Properties of Plant Polysaccharides. Am. J. Chin. Med..

[45] Ge Y.Z., Ahmed S., Yao W.Z., You L.J., Zheng J.X., Hileuskaya K. (2021). Regulation effects of indigestible dietary polysaccharides on intestinal microflora: An overview. J. Food Biochem..

[46] Xu X.F., Xu P.P., Ma C.W., Tang J., Zhang X.W. (2013). Gut microbiota, host health, and polysaccharides. Biotechnol. Adv..

[47] Cai Y., Liu W., Lin Y.X., Zhang S.B., Zou B.R., Xiao D., Lin L., Zhong Y.P., Zheng H.H., Liao Q.F. (2019). Compound polysaccharides ameliorate experimental colitis by modulating gut microbiota composition and function. J. Gastroenterol. Hepatol..

[48] Bo R., Liu Z., Zhang J., Gu P., Ou N., Sun Y., Hu Y., Liu J., Wang D. (2019). Mechanism of Lycium barbarum polysaccharides liposomes on activating murine dendritic cells. Carbohydr. Polym..

[49] Chen N., Zhang H., Zong X., Li S., Wang J., Wang Y., Jin M. (2020). Polysaccharides from Auricularia auricula: Preparation, structural features and biological activities. Carbohydr. Polym..

[50] Gong P.X., Wu Y.C., Liu Y., Lv S.Z., You Y., Zhou Z.L., Chen X., Li H.J. (2022). Structure and hypoglycemic effect of a neutral polysaccharide isolated from sea cucumber Stichopus japonicus. Int. J. Biol. Macromol..

[51] Guidara M., Yaich H., Amor I.B., Fakhfakh J., Gargouri J., Lassoued S., Blecker C., Richel A., Attia H., Garna H. (2021). Effect of extraction procedures on the chemical structure, antitumor and anticoagulant properties of ulvan from Ulva lactuca of Tunisia coast. Carbohydr. Polym..

[52] Kumar A., Rao K.M., Han S.S. (2018). Application of xanthan gum as polysaccharide in tissue engineering: A review. Carbohydr. Polym..

[53] Svensson M.V., Zhang X., Huttunen E., Widmalm G. (2011). Structural studies of the capsular polysaccharide produced by Leuconostoc mesenteroides ssp. cremoris PIA2. Biomacromolecules.

[54] Teng S., Zhang Y., Jin X., Zhu Y., Li L., Huang X., Wang D., Lin Z. (2023). Structure and hepatoprotective activity of Usp10/NF-kappaB/Nrf2 pathway-related Morchella esculenta polysaccharide. Carbohydr. Polym..

[55] Yuan Q.H., Xie F., Tan J., Yuan Y., Mei H., Zheng Y., Sheng R. (2022). Extraction, structure and pharmacological effects of the polysaccharides from Cordyceps sinensis: A review. J. Funct. Foods.

[56] Zhou Y., Chen X.X., Chen T.T., Chen X.Q. (2022). A review of the antibacterial activity and mechanisms of plant polysaccharides. Trends Food Sci. Tech..

[57] Zhou J.N., Li R.L., Jia Y.A., Wang Y.J., Liu J.Y., Panichayupakaranant P., Chen H.X. (2022). Recent Progress in Natural Anticancer Agents Discovery from Tea (Camellia sinensis): A Review. Recent. Pat. Anti-Cancer.

[58] Zhu J.X., Chen Z.Y., Zhou H., Yu C., Han Z., Shao S.R., Hu X.C., Wei X.L., Wang Y.F. (2020). Effects of extraction methods on physicochemical properties and hypoglycemic activities of polysaccharides from coarse green tea. Glycoconj. J..

[59] Alba K., Offiah V., Laws A.P., Falade K.O., Kontogiorgos V. (2020). Baobab polysaccharides from fruits and leaves. Food Hydrocoll..

[60] Xu L.L., Chen Y., Chen Z.Q., Gao X.D., Wang C.L., Panichayupakaranant P., Chen H.X. (2020). Ultrafiltration isolation, physicochemical characterization, and antidiabetic activities analysis of polysaccharides from green tea, oolong tea, and black tea. J. Food Sci..

[61] Guilbaud A., Howsam M., Niquet-Léridon C., Delguste F., Fremont M., Lestavel S., Maboudou P., Garat A., Schraen S., Onraed B. (2020). The Effect of Lactobacillus fermentum ME-3 Treatment on Glycation and Diabetes Complications. Mol. Nutr. Food Res..

[62] Bao Y., Zhang Y.C., Zhang Y., Liu Y., Wang S.Q., Dong X.M., Wang Y.Y., Zhang H.P. (2010). Screening of potential probiotic properties of Lactobacillus fermentum isolated from traditional dairy products. Food Control.

[63] Saavedra J.M., Bauman N.A., Perman J.A., Yolken R.H., Saavedra J.M., Bauman N.A., Oung I. (1994). Feeding of Bifidobacterium bifidum and Streptococcus thermophilus to infants in hospital for prevention of diarrhoea and shedding of rotavirus. Lancet.

[64] Makras L., Triantafyllou V., Fayol-Messaoudi D., Adriany T., Zoumpopoulou G., Tsakalidou E., Servin A., De Vuyst L. (2006). Kinetic analysis of the antibacterial activity of probiotic lactobacilli towards Salmonella enterica serovar Typhimurium reveals a role for lactic acid and other inhibitory compounds. Res. Microbiol..

[65] Chen X.Y., Tan F., Yi R.K., Mu J.F., Zhao X., Yang Z.N. (2018). Effects of Lactobacillus on Mice with Diabetes Induced by High-Fat Diet with Streptozotocin (STZ). Appl. Sci..

[66] Din A.U., Hassan A., Zhu Y., Zhang K., Wang Y., Li T., Wang Y., Wang G. (2020). Inhibitory effect of Bifidobacterium bifidum ATCC 29521 on colitis and its mechanism. J. Nutr. Biochem..

[67] Fernando W.M.A.D.B., Flint S.H., Ranaweera K.K.D.S., Bamunuarachchi A., Johnson S.K., Brennan C.S. (2018). The potential synergistic behaviour of inter- and intra-genus probiotic combinations in the pattern and rate of short chain fatty acids formation during fibre fermentation. Int. J. Food Sci. Nutr..

[68] Chen M., Xiao D., Liu W., Song Y., Zou B., Li L., Li P., Cai Y., Liu D., Liao Q. (2020). Intake of Ganoderma lucidum polysaccharides reverses the disturbed gut microbiota and metabolism in type 2 diabetic rats. Int. J. Biol. Macromol..

[69] Li S., Qi Y., Chen L., Qu D., Li Z., Gao K., Chen J., Sun Y. (2019). Effects of Panax ginseng polysaccharides on the gut microbiota in mice with antibiotic-associated diarrhea. Int. J. Biol. Macromol..

[70] Yang L.N., Huang J.H., Wu X.H., Li L., Cai W.Q., Zhu L.J., Wang S.N., Song H., Zhu D.S., Ma T. (2021). Interactions between gut microbiota and soy hull polysaccharides regulate the air-liquid interfacial activity. Food Hydrocoll..

[71] Turroni F., Milani C., Duranti S., Mahony J., van Sinderen D., Ventura M. (2018). Glycan Utilization and Cross-Feeding Activities by Bifidobacteria. Trends Microbiol..

[72] Wang H.Y., Guo L.X., Hu W.H., Peng Z.T., Wang C., Chen Z.C., Liu E.Y.L., Dong T.T.X., Wang T.J., Tsim K.W.K. (2019). Polysaccharide from tuberous roots of Ophiopogon japonicus regulates gut microbiota and its metabolites during alleviation of high-fat diet-induced type-2 diabetes in mice. J. Funct. Foods.

[73] Li J., Pang B., Yan X., Shang X., Hu X., Shi J. (2020). Prebiotic properties of different polysaccharide fractions from Artemisia sphaerocephala Krasch seeds evaluated by simulated digestion and in vitro fermentation by human fecal microbiota. Int. J. Biol. Macromol..

[74] Yan H.L., Lu J.M., Wang Y.F., Gu W., Yang X.X., Yu J. (2017). Intake of total saponins and polysaccharides from Polygonatum kingianum affects the gut microbiota in diabetic rats. Phytomedicine.

[75] Liang J.J., Zhang M.N., Wang X.N., Ren Y.C., Yue T.L., Wang Z.L., Gao Z.P. (2021). Edible fungal polysaccharides, the gut microbiota, and host health. Carbohydr. Polym..

[76] Yang L.A., Li L., Wu X.H., Cai W.Q., Lin Q., Zhu D.S. (2021). The Effect of Natural Soluble Polysaccharides on the Type 2 Diabetes through Modulating Gut Microbiota: A Review. Curr. Med. Chem..

[77] Rehman A.U., Khan A.I., Xin Y., Liang W. (2022). Morchella esculenta polysaccharide attenuate obesity, inflammation and modulate gut microbiota. Amb. Express.

[78] Ren F., Meng C., Chen W.J., Chen H.M., Chen W.X. (2021). Ganoderma amboinense polysaccharide prevents obesity by regulating gut microbiota in high-fat-diet mice. Food Biosci..

[79] Su A.X., Ma G.X., Ma N., Pei F., Yang W.J., Hu Q.H. (2023). Effects of Flammulina velutipes polysaccharides on gut microbiota composition and metabolism in vitro fermentation. Food Sci. Biotechnol..

[80] Zhong L., Ma N., Zheng H.H., Ma G.X., Zhao L.Y., Hu Q.H. (2019). Tuber indicum polysaccharide relieves fatigue by regulating gut microbiota in mice. J. Funct. Foods.

[81] Yu R.X., Luo J.M., Liu L., Peng X.C., Gan R.Y., Wu D.T., Hu Y.C. (2024). Hypoglycemic Effect of Edible Fungi Polysaccharides Depends on Their Metabolites from the Fermentation of Human Fecal Microbiota. Foods.

[82] Fuller R., Moore M.V., Lewith G., Stuart B.L., Ormiston R.V., Fisk H.L., Noakes P.S., Calder P.C. (2017). Yeast-derived β-1,3/1,6 glucan, upper respiratory tract infection and innate immunity in older adults. Nutrition.

[83] Gudi R., Perez N., Johnson B.M., Sofi M.H., Brown R., Quan S., Karumuthil-Melethil S., Vasu C. (2019). Complex dietary polysaccharide modulates gut immune function and microbiota, and promotes protection from autoimmune diabetes. Immunology.

[84] Guo W.L., Deng J.C., Pan Y.Y., Xu J.X., Hong J.L., Shi F.F., Liu G.L., Qian M., Bai W.D., Zhang W. (2020). Hypoglycemic and hypolipidemic activities of Grifola frondosa polysaccharides and their relationships with the modulation of intestinal microflora in diabetic mice induced by high-fat diet and streptozotocin. Int. J. Biol. Macromol..

[85] Ouyang Y.Z., Nie J.P., Gao X.X., Zhao C. (2022). Grifola Frondosa Polysaccharide Ameliorates Hyperglycemia and Gut Microbiota in Type 2 Diabetic Mice. Free Radic. Biol. Med..

[86] Fu C.J., Ye K., Ma S., Du H.J., Chen S.G., Liu D.H., Ma G.X., Xiao H. (2023). Simulated gastrointestinal digestion and gut microbiota fermentation of polysaccharides from Agaricus bisporus. Food Chem..

[87] Tsung-Ru W., Chuan-Sheng L., Chih-Jung C., Tzu-Lung L., Jan M., Yun-Fei K., David M.O., Chia-Chen L., John D.Y., Hsin-Chih L. (2019). Gut commensal Parabacteroides goldsteiniiplays plays a predominant role in the anti-obesity effects of polysaccharides isolated from Hirsutella sinensis. Gut.

[88] Chen X.X., Chen C., Fu X. (2022). Hypoglycemic effect of the polysaccharides from Astragalus membranaceus on type 2 diabetic mice based on the “gut microbiota-mucosal barrier”. Food Funct..

[89] Zhou W., Han L.J., Raza S.H.A., Yue Q.M., Sun S.N., Zhao Y.X., Lv L.F., Deng Y.R., Yuan Z.Z., Alsharif I. (2023). Polysaccharides in Berberis dasystachya improve intestinal flora depending on the molecular weight and ameliorate type 2 diabetes in rats. J. Funct. Foods.

[90] Liu G.D., Feng S.M., Yan J.D., Luan D., Sun P.L., Shao P. (2022). Antidiabetic potential of polysaccharides from Brasenia schreberi regulating insulin signaling pathway and gut microbiota in type 2 diabetic mice. Curr. Res. Food Sci..

[91] Li H.S., Fang Q.Y., Nie Q.X., Hu J.L., Yang C., Huang T., Li H., Nie S.P. (2020). Hypoglycemic and Hypolipidemic Mechanism of Tea Polysaccharides on Type 2 Diabetic Rats via Gut Microbiota and Metabolism Alteration. J. Agric. Food Chem..

[92] Xia T., Liu C.S., Hu Y.N., Luo Z.Y., Chen F.L., Yuan L.X., Tan X.M. (2021). Coix seed polysaccharides alleviate type 2 diabetes mellitus via gut microbiota-derived short-chain fatty acids activation of IGF1/PI3K/ AKT signaling. Food Res. Int..

[93] Ma Q.Y., Zhai R.H., Xie X.Q., Chen T., Zhang Z.Q., Liu H.C., Nie C.X., Yuan X.J., Tu A.B., Tian B.M. (2022). Hypoglycemic Effects of Lycium barbarum Polysaccharide in Type 2 Diabetes Mellitus Mice Modulating Gut Microbiota. Front. Nutr..

[94] Dong J., Liang Q.X., Niu Y., Jiang S.J., Zhou L., Wang J.M., Ma C.Y., Kang W.Y. (2020). Effects of Nigella sativa seed polysaccharides on type 2 diabetic mice and gut microbiota. Int. J. Biol. Macromol..

[95] Zhang M., Yang L.C., Zhu M.M., Yang B., Yang Y.J., Jia X.B., Feng L. (2022). Moutan Cortex polysaccharide ameliorates diabetic kidney disease via modulating gut microbiota dynamically in rats. Int. J. Biol. Macromol..

[96] Xu N., Zhou Y.J., Lu X.Y., Chang Y.N. (2021). Auricularia auricula-judae (Bull.) polysaccharides improve type 2 diabetes in HFD/STZ-induced mice by regulating the AKT/AMPK signaling pathways and the gut microbiota. J. Food Sci..

[97] Shao W.M., Xiao C., Yong T.Q., Zhang Y.F., Hu H.P., Xie T., Liu R.J., Huang L.H., Li X.M., Xie Y.Z. (2022). A polysaccharide isolated from Ganoderma lucidum ameliorates hyperglycemia through modulating gut microbiota in type 2 diabetic mice. Int. J. Biol. Macromol..

[98] Zhao H., Li M., Liu L., Li D., Zhao L., Wu Z., Zhou M., Jia L., Yang F. (2023). Cordyceps militaris polysaccharide alleviates diabetic symptoms by regulating gut microbiota against TLR4/NF-κB pathway. Int. J. Biol. Macromol..

[99] Chen Y.Q., Liu D., Wang D.Y., Lai S.S., Zhong R.T., Liu Y.Y., Yang C.F., Liu B., Sarker M.R., Zhao C. (2019). Hypoglycemic activity and gut microbiota regulation of a novel polysaccharide from Grifola frondosa in type 2 diabetic mice. Food Chem. Toxicol..

[100] Rehman A.U., Siddiqui N.Z., Farooqui N.A., Alam G., Gul A., Ahmad B., Asim M., Khan A.I., Xin Y., Zexu W. (2022). Morchella esculenta mushroom polysaccharide attenuates diabetes and modulates intestinal permeability and gut microbiota in a type 2 diabetic mice model. Front. Nutr..

[101] Xue Z.H., Li R.L., Liu J.Y., Zhou J.N., Zhang X.Y., Zhang T.T., Zhang M., Yang Y., Chen H.X. (2023). Preventive and synbiotic effects of the soluble dietary fiber obtained from Lentinula edodes byproducts and Lactobacillus plantarum against dextran sulfate sodium-induced colitis in mice. J. Sci. Food Agric..

[102] Kang N., Oh S., Kim S.Y., Ahn H., Son M., Heo S.J., Byun K., Jeon Y.J. (2022). Anti-obesity effects of Ishophloroglucin A from the brown seaweed Ishige okamurae (Yendo) regulation of leptin signal in ob/ob mice. Algal Res..

[103] Lee S.H., Kim M., Park M.H. (2021). Diphlorethohydroxycamalol isolated from Ishige okamurae prevents H2O2-induced oxidative damage via BMP2/Runx2 signaling in osteoblastic MC3T3-E1 cells. Fitoterapia.

[104] Xiao Z.B., Yang S.T., Liu Y., Zhou C.X., Hong P.Z., Sun S.L., Qian Z.J. (2022). A novel glyceroglycolipid from brown algae Ishige okamurae improve photoaging and counteract inflammation in UVB-induced HaCaT cells. Chem.-Biol. Interact..

[105] Yang H.W., Fernando K.H.N., Oh J.Y., Li X., Jeon Y.J., Ryu B. (2019). Anti-Obesity and Anti-Diabetic Effects of Ishige okamurae. Mar. Drugs.

[106] Usoltseva R.V., Anastyuk S.D., Shevchenko N.M., Surits V.V., Silchenko A.S., Isakov V.V., Zvyagintseva T.N., Thinh P.D., Ermakova S.P. (2017). Polysaccharides from brown algae Sargassum duplicatum: The structure and anticancer activity. Carbohydr. Polym..

[107] Zhong Q.W., Zhou T.S., Qiu W.H., Wang Y.K., Xu Q.L., Ke S.Z., Wang S.J., Jin W.H., Chen J.W., Zhang H.W. (2021). Characterization and hypoglycemic effects of sulfated polysaccharides derived from brown seaweed. Food Chem..

[108] Park J., Cha J.D., Choi K.M., Lee K.Y., Han K.M., Jang Y.S. (2017). Fucoidan inhibits LPS-induced inflammation in vitro and during the acute response in vivo. Int. Immunopharmacol..

[109] Han R., Pang D.R., Wen L.R., You L.J., Huang R.M., Kulikouskaya V. (2020). In vitro digestibility and prebiotic activities of a sulfated polysaccharide from Gracilaria Lemaneiformis. J. Funct. Foods.

[110] Mou J.J., Li Q., Shi W.W., Qi X.H., Song W.G., Yang J. (2020). Chain conformation, physicochemical properties of fucosylated chondroitin sulfate from sea cucumber Stichopus chloronotus and its in vitro fermentation by human gut microbiota. Carbohydr. Polym..

[111] Seong H., Bae J.H., Seo J.S., Kim S.A., Kim T.J., Han N.S. (2019). Comparative analysis of prebiotic effects of seaweed polysaccharides laminaran, porphyran, and ulvan using in vitro human fecal fermentation. J. Funct. Foods.

[112] Pratap K., Majzoub M.E., Taki A.C., Hernandez S.M., Magnusson M., Glasson C.R.K., de Nys R., Thomas T., Lopata A.L., Kamath S.D. (2022). The Algal Polysaccharide Ulvan and Carotenoid Astaxanthin Both Positively Modulate Gut Microbiota in Mice. Foods.

[113] Liu Z.Q., Yan C.H., Lin X.P., Ai C.Q., Dong X.P., Shao L., Wang S.T., Song S., Zhu B.W. (2022). Responses of the gut microbiota and metabolite profiles to sulfated polysaccharides from sea cucumber in humanized microbiota mice. Food Funct..

[114] Zhu Z.J., Zhu B.W., Sun Y.J., Ai C.Q., Wang L.L., Wen C.R., Yang J.F., Song S., Liu X.L. (2018). Sulfated Polysaccharide from Sea Cucumber and its Depolymerized Derivative Prevent Obesity in Association with Modification of Gut Microbiota in High-Fat Diet-Fed Mice. Mol. Nutr. Food Res..

[115] Wei B., Zhong Q.-W., Ke S.-Z., Zhou T.-S., Xu Q.-L., Wang S.-J., Chen J.-W., Zhang H.-W., Jin W.-H., Wang H. (2020). Sargassum fusiforme Polysaccharides Prevent High-Fat Diet-Induced Early Fasting Hypoglycemia and Regulate the Gut Microbiota Composition. Mar. Drugs.

[116] Lin H.T., Zhang J.W., Li S.Y., Zheng B.D., Hu J.M. (2021). Polysaccharides isolated from Laminaria japonica attenuates gestational diabetes mellitus by regulating the gut microbiota in mice. Food Front..

[117] Siddiqui N.Z., Rehman A.U., Yousuf W., Khan A.I., Farooqui N.A., Zang S.Z., Xin Y., Wang L. (2022). Effect of crude polysaccharide from seaweed, Dictyopteris divaricata (CDDP) on gut microbiota restoration and anti-diabetic activity in streptozotocin (STZ)-induced T1DM mice. Gut Pathog..

[118] Zhao F.Q., Liu Q.B., Cao J., Xu Y.S., Pei Z.S., Fan H.F., Yuan Y.Q., Shen X.R., Li C. (2020). A sea cucumber (Holothuria leucospilota) polysaccharide improves the gut microbiome to alleviate the symptoms of type 2 diabetes mellitus in Goto-Kakizaki rats. Food Chem. Toxicol..

[119] Ruan Q.L., Chen Y.H., Wen J.H., Qiu Y.H., Huang Y.J., Zhang Y., Farag M.A., Zhao C. (2023). Regulatory mechanisms of the edible alga Ulva lactuca polysaccharide via modulation of gut microbiota in diabetic mice. Food Chem..

[120] Jia R.B., Li Z.R., Lin L.Z., Luo D.H., Chen C., Zhao M.M. (2022). The potential mechanisms of Macrocystis pyrifera polysaccharides mitigating type 2 diabetes in rats. Food Funct..

[121] Zhao Y.F., Song P.L., Yin S., Fan T.Y., Li F.W., Ge X.D., Liu T.T., Xu W., Xu S., Chen L.G. (2023). Onchidium struma polysaccharides exhibit hypoglycemic activity and modulate the gut microbiota in mice with type 2 diabetes mellitus. Food Funct..

[122] Zhao L.P., Zhang F., Ding X.Y., Wu G.J., Lam Y.Y., Wang X.J., Fu H.Q., Xue X.H., Lu C.H., Ma J.L. (2018). Gut bacteria selectively promoted by dietary fibers alleviate type 2 diabetes. Science.

[123] Sun Z.Y., Yu S., Tian Y., Han B.Q., Zhao Y., Li Y.Q., Wang Y., Sun Y.J., Shen W. (2022). Chestnut polysaccharides restore impaired spermatogenesis by adjusting gut microbiota and the intestinal structure. Food Funct..

[124] Martínez-López Y.E., Esquivel-Hernández D.A., Sánchez-Castañeda J.P., Neri-Rosario D., Guardado-Mendoza R., Resendis-Antonio O. (2022). Type 2 diabetes, gut microbiome, and systems biology: A novel perspective for a new era. Gut Microbes.

[125] Yang J.P., Summanen P.H., Henning S.M., Hsu M., Lam H., Huang J.J., Tseng C.H., Dowd S.E., Finegold S.M., Heber D. (2015). Xylooligosaccharide supplementation alters gut bacteria in both healthy and prediabetic adults: A pilot study. Front. Physiol..

[126] Santilli A., Stefanopoulos S., Cresci G.A.M. (2022). The gut barrier and chronic diseases. Curr. Opin. Clin. Nutr..

[127] Wang Y.J., Chen Y., Zhang X.Y., Lu Y.P., Chen H.X. (2020). New insights in intestinal oxidative stress damage and the health intervention effects of nutrients: A review. J. Funct. Foods.

[128] Fang J.Y., Lin Y., Xie H.L., Farag M.A., Feng S.M., Li J.J., Shao P. (2022). Dendrobium officinale leaf polysaccharides ameliorated hyperglycemia and promoted gut bacterial associated SCFAs to alleviate type 2 diabetes in adult mice. Food Chem. X.

[129] Macho-González A., Garcimartín A., Redondo N., Cofrades S., Bastida S., Nova E., Benedí J., Sánchez-Muniz F.J., Marcos A., López-Oliva M.E. (2021). Carob fruit extract-enriched meat, as preventive and curative treatments, improves gut microbiota and colonic barrier integrity in a late-stage T2DM model. Food Res. Int..

[130] Ma G.X., Ma S., Du H.J., Li X.Y., Tao Q., Hu Q.H., Xiao H. (2024). Interactions between intestinal microbial fermentation products of Pleurotus eryngii polysaccharide with gut mucus. Food Funct..

[131] Jiang G.Y., Lei A.T., Chen Y., Yu Q., Xie J.H., Yang Y., Yuan T.J., Su D. (2021). The protective effects of the Ganoderma atrum polysaccharide against acrylamide-induced inflammation and oxidative damage in rats. Food Funct..

[132] Bai Y.F., Yue Z.L., Wang Y.N., Li Y.D., Li C., Liu X.T., Shi R.H., Huo N.N., Li D.D., Gao S. (2023). Synergistic effect of polysaccharides and flavonoids on lipid and gut microbiota in hyperlipidemic rats. Food Funct..

[133] Li Z., Li X.Y., Shi P.P., Li P.P., Fu Y., Tan G.F., Zhou J.J., Zeng J.G., Huang P. (2024). Modulation of Acute Intestinal Inflammation by Dandelion Polysaccharides: An In-Depth Analysis of Antioxidative, Anti-Inflammatory Effects and Gut Microbiota Regulation. Int. J. Mol. Sci..

[134] Liu Y.Y., Wang C.R., Li J.S., Li T.T., Zhang Y., Liang Y.X., Mei Y.X. (2020). Phellinus linteus polysaccharide extract improves insulin resistance by regulating gut microbiota composition. FASEB J..

[135] van Muijlwijk G.H., van Mierlo G., Jansen P.W.T.C., Vermeulen M., Bleumink-Pluym N.M.C., Palm N.W., van Putten J.P.M., de Zoete M.R. (2021). Identification of Allobaculum mucolyticum as a novel human intestinal mucin degrader. Gut Microbes.

[136] Zhou W.T., Yang T.T., Xu W.Q., Huang Y.J., Ran L.W., Yan Y.M., Mi J., Lu L., Sun Y., Zeng X.X. (2022). The polysaccharides from the fruits of Lycium barbarum L. confer anti-diabetic effect by regulating gut microbiota and intestinal barrier. Carbohydr. Polym..

[137] Cordeiro L.M.C., Reinhardt V.D., Baggio C.H., Werner M.F.D., Burci L.M., Sassaki G.L., Iacomini M. (2012). Arabinan and arabinan-rich pectic polysaccharides from quinoa (Chenopodium quinoa) seeds: Structure and gastroprotective activity. Food Chem..

[138] Wang M.X., Chen Y.X., Wang Y.Y., Li Y., Zheng H.H., Ma F.L., Ma C.W., Zhang X.J., Lu B.Y., Xie Z.Y. (2018). The effect of probiotics and polysaccharides on the gut microbiota composition and function of weaned rats. Food Funct..

[139] Guo C.L., Zhang S.H., Wang Y.Q., Li M.X., Ding K. (2020). Isolation and structure characterization of a polysaccharide from Crataegus pinnatifida and its bioactivity on gut microbiota. Int. J. Biol. Macromol..

[140] Song Q.B., Cheng S.W., Li D., Cheng H.Y., Lai Y.S., Han Q.B., Wu H.Y., Shaw P.C., Zuo Z. (2022). Gut microbiota mediated hypoglycemic effect of Astragalus membranaceus polysaccharides in db/db mice. Front. Pharmacol..

[141] Granado-Serrano A.B., Martín-Garí M., Sánchez V., Solans M.R., Berdún R., Ludwig I.A., Rubió L., Vilaprinyó E., Portero-Otín M., Serrano J.C.E. (2019). Faecal bacterial and short-chain fatty acids signature in hypercholesterolemia. Sci. Rep..

[142] Huang Y.Z., Chen H., Zhang K.F., Lu Y.M., Wu Q.Z., Chen J.L., Li Y., Wu Q.X., Chen Y. (2022). Extraction, purification, structural characterization, and gut microbiota relationship of polysaccharides: A review. Int. J. Biol. Macromol..

[143] Soverini M., Turroni S., Biagi E., Quercia S., Brigidi P., Candela M., Rampelli S. (2017). Variation of Carbohydrate-Active Enzyme Patterns in the Gut Microbiota of Italian Healthy Subjects and Type 2 Diabetes Patients. Front. Microbiol..

[144] Li Q., Hu J., Nie Q., Chang X., Fang Q., Xie J., Li H., Nie S. (2021). Hypoglycemic mechanism of polysaccharide from Cyclocarya paliurus leaves in type 2 diabetic rats by gut microbiota and host metabolism alteration. Sci. China Life Sci..

[145] Deng J., Zhong J., Long J., Zou X.Y., Wang D., Song Y., Zhou K., Liang Y.X., Huang R.M., Wei X.Q. (2020). Hypoglycemic effects and mechanism of different molecular weights of konjac glucomannans in type 2 diabetic rats. Int. J. Biol. Macromol..

[146] Zhong L., Peng X.J., Wu C.T., Li Q., Chen Y.F., Wang M., Li Y.T., He K.Y., Shi Y., Bie C.Q. (2023). Polysaccharides and flavonoids from cyclocarya paliurus modulate gut microbiota and attenuate hepatic steatosis, hyperglycemia, and hyperlipidemia in nonalcoholic fatty liver disease rats with type 2 diabetes mellitus. Int. J. Diabetes Dev. Ctries..

[147] Collado M.C., Derrien M., Isolauri E. (2007). Intestinal integrity and Akkermansia muciniphila, a mucin-degrading member of the intestinal microbiota present in infants, adults, and the elderly. Appl. Environ. Microb..

[148] Shin N.R., Lee J.C., Lee H.Y., Kim M.S., Whon T.W., Lee M.S., Bae J.W. (2014). An increase in the Akkermansia spp. population induced by metformin treatment improves glucose homeostasis in diet-induced obese mice. Gut.

[149] Deng Q.H., Wang W.J., Zhang L.Y., Chen L.L., Zhang Q.F., Zhang Y., He S.C., Li J.E. (2023). Gougunao tea polysaccharides ameliorate high-fat diet-induced hyperlipidemia and modulate gut microbiota. Food Funct..

[150] Huo J.Y., Lei M., Li F.F., Hou J.J., Zhang Z.J., Long H.L., Zhong X.C., Liu Y.M., Xie C., Wu W.Y. (2021). Structural Characterization of a Polysaccharide from Gastrodia elata and Its Bioactivity on Gut Microbiota. Molecules.

[151] Wu H.Q., Ma Z.L., Zhang D.X., Wu P., Guo Y.H., Yang F., Li D.Y. (2021). Sequential Extraction, Characterization, and Analysis of Pumpkin Polysaccharides for Their Hypoglycemic Activities and Effects on Gut Microbiota in Mice. Front. Nutr..

[152] Fu Z.F., Han L.F., Zhang P., Mao H.P., Zhang H., Wang Y.F., Gao X.M., Liu E.W. (2020). Cistanche polysaccharides enhance echinacoside absorption in vivo and affect the gut microbiota. Int. J. Biol. Macromol..

[153] Milton-Laskibar I., Cuevas-Sierra A., Portillo M.P., Martínez J.A. (2022). Effects of Resveratrol Administration in Liver Injury Prevention as Induced by an Obesogenic Diet: Role of Ruminococcaceae. Biomedicines.

[154] Li L., Guo W.L., Zhang W., Xu J.X., Qian M., Bai W.D., Zhang Y.Y., Rao P.F., Ni L., Lv X.C. (2019). Grifola frondosa polysaccharides ameliorate lipid metabolic disorders and gut microbiota dysbiosis in high-fat diet fed rats. Food Funct..

[155] Dziarski R., Park S.Y., Kashyap D.R., Dowd S.E., Gupta D. (2016). Pglyrp-Regulated Gut Microflora Prevotella falsenii, Parabacteroides distasonis and Bacteroides eggerthii Enhance and Attenuates Colitis in Mice. PLoS ONE.

[156] Gao X.X., Liu D., Gao L.Y., Ouyang Y.Z., Wen Y.X., Ai C., Chen Y.Q., Zhao C. (2022). Health benefits of Grifola frondosa polysaccharide on intestinal microbiota in type 2 diabetic mice. Food Sci. Hum. Well.

[157] Xu X.F., Zhang X.W. (2015). Lentinula edodes-Derived Polysaccharide Alters the Spatial Structure of Gut Microbiota in Mice. PLoS ONE.

[158] Liu Q., An X., Chen Y., Deng Y.X., Niu H.L., Ma R.S., Zhao H.A., Cao W., Wang X.R., Wang M. (2022). Effects of Auricularia auricula Polysaccharides on Gut Microbiota and Metabolic Phenotype in Mice. Foods.

[159] Su L., Xin C.X., Yang J.T., Dong L.R., Mei H.R.B., Dai X.J., Wang Q. (2022). A polysaccharide from Inonotus obliquus ameliorates intestinal barrier dysfunction in mice with type 2 diabetes mellitus. Int. J. Biol. Macromol..

[160] Khan I., Huang G.X., Li X.A., Leong W., Xia W.R., Hsiao W.L.W. (2018). Mushroom polysaccharides from Ganoderma lucidum and Poria cocos reveal prebiotic functions. J. Funct. Foods.

[161] Christovich A., Luo X.M. (2022). Gut Microbiota, Leaky Gut, and Autoimmune Diseases. Front. Immunol..

[162] Richards J.L., Yap Y.A., McLeod K.H., Mackay C.R., Mariño E. (2016). Dietary metabolites and the gut microbiota: An alternative approach to control inflammatory and autoimmune diseases. Clin. Transl. Immunol..

[163] Wang Y., Sun M.Y., Jin H.Y., Yang J.B., Kang S., Liu Y., Yang S., Ma S.C., Ni J. (2021). Effects of Lycium barbarum Polysaccharides on Immunity and the Gut Microbiota in Cyclophosphamide-Induced Immunosuppressed Mice. Front. Microbiol..

[164] Tan T.G., Sefik E., Geva-Zatorsky N., Kua L., Naskar D., Teng F., Pasman L., Ortiz-Lopez A., Jupp R., Wu H.J.J. (2016). Identifying species of symbiont bacteria from the human gut that, alone, can induce intestinal Th17 cells in mice. Proc. Natl. Acad. Sci. USA.

[165] Bunker J.J., Drees C., Watson A.R., Plunkett C.H., Nagler C.R., Schneewind O., Eren A.M., Bendelac A. (2019). B cell superantigens in the human intestinal microbiota. Sci. Transl. Med..

[166] Shin N.R., Whon T.W., Bae J.W. (2015). Proteobacteria: Microbial signature of dysbiosis in gut microbiota. Trends Biotechnol..

[167] Zhao M.Q., Tang F., Huang X.Y., Ma J.J., Wang F.M., Zhang P. (2024). Polysaccharide Isolated from Agaricus blazei Murill Alleviates Intestinal Ischemia/Reperfusion Injury through Regulating Gut Microbiota and Mitigating Inflammation in Mice. J. Agric. Food Chem..

[168] Xu T., Ge Y.M., Du H., Li Q., Xu X.M., Yi H., Wu X.Y., Kuang T.T., Fan G., Zhang Y. (2021). Berberis kansuensis extract alleviates type 2 diabetes in rats by regulating gut microbiota composition. J. Ethnopharmacol..

[169] Xia W., Li X., Su L., Khan I., Leong W.K., Yin L., Bian X., Su J., Huang G., Hsiao W.L.W. (2020). Corrigendum to “Lycium Berry Polysaccharides Strengthen Gut Microenvironment and Modulate Gut Microbiota of the Mice”. Evid. Based Complement. Altern. Med..

[170] Zhang F.H., Wang M., Yang J.J., Xu Q., Liang C., Chen B., Zhang J.M., Yang Y., Wang H.L., Shang Y.F. (2019). Response of gut microbiota in type 2 diabetes to hypoglycemic agents. Endocrine.

[171] Liu X., Zhang J.J., Li Y.M., Sun L.Y., Xiao Y., Gao W.G., Zhang Z.S. (2019). Mogroside derivatives exert hypoglycemics effects by decreasing blood glucose level in HepG2 cells and alleviates insulin resistance in T2DM rats. J. Funct. Foods.

[172] Jiang Q.H., Chen L., Wang R., Chen Y., Deng S.G., Shen G.X., Liu S.L., Xiang X.W. (2024). Hypoglycemic mechanism of Tegillarca granosa polysaccharides on type 2 diabetic mice by altering gut microbiota and regulating the PI3K-akt signaling pathway. Food Sci. Hum. Well.

[173] Zhong R.T., Chen L.B., Liu Y.Y., Xie S.X., Li S.M., Liu B., Zhao C. (2022). Anti-diabetic effect of aloin via JNK-IRS1/PI3K pathways and regulation of gut microbiota. Food Sci. Hum. Well.

[174] Li D., Feng G.L., Li Y., Pan H., Luo P., Liu B., Ding T., Wang X., Xu H.B., Zhao Y.F. (2023). Benefits of Huang Lian mediated by gut microbiota on HFD/STZ-induced type 2 diabetes mellitus in mice. Front. Endocrinol..

[175] Wu G.J., Bai Z.Y., Wan Y.J., Shi H.F., Huang X.J., Nie S.P. (2020). Antidiabetic effects of polysaccharide from azuki bean (Vigna angularis) in type 2 diabetic rats via insulin/PI3K/AKT signaling pathway. Food Hydrocoll..

[176] Yang C.F., Lai S.S., Chen Y.H., Liu D., Liu B., Ai C., Wan X.Z., Gao L.Y., Chen X.H., Zhao C. (2019). Anti-diabetic effect of oligosaccharides from seaweed Sargassum confusum via JNK-IRS1/PI3K signalling pathways and regulation of gut microbiota. Food Chem. Toxicol..

[177] Lu H., Liu P., Zhang X., Bao T., Wang T., Guo L., Li Y., Dong X., Li X., Dong Y. (2021). Inulin and Lycium barbarum polysaccharides ameliorate diabetes by enhancing gut barrier via modulating gut microbiota and activating gut mucosal TLR2+ intraepithelial γδ T cells in rats. J. Funct. Foods.

[178] Zhang C.H., Sheng J.Q., Sarsaiya S., Shu F.X., Liu T.T., Tu X.Y., Ma G.Q., Xu G.L., Zheng H.X., Zhou L.F. (2019). The anti-diabetic activities, gut microbiota composition, the anti-inflammatory effects of Scutellaria-coptis herb couple against insulin resistance-model of diabetes involving the toll-like receptor 4 signaling pathway. J. Ethnopharmacol..

[179] Fernandez-Quintela A., Macarulla M.T., Gómez-Zorita S., González M., Milton-Laskibar I., Portillo M.P. (2023). Relationship between changes in microbiota induced by resveratrol and its anti-diabetic effect on type 2 diabetes. Front. Nutr..

[180] Chassaing B., Etienne-Mesmin L., Gewirtz A.T. (2014). Microbiota-Liver Axis in Hepatic Disease. Hepatology.

[181] de Clercq N.C., Frissen M.N., Groen A.K., Nieuwdorp M. (2017). Gut Microbiota and the Gut-Brain Axis: New Insights in the Pathophysiology of Metabolic Syndrome. Psychosom. Med..

[182] Dumas A., Bernard L., Poquet Y., Lugo-Villarino G., Neyrolles O. (2018). The role of the lung microbiota and the gut-lung axis in respiratory infectious diseases. Cell Microbiol..

[183] Huang Y.H., Xin W., Xiong J.C., Yao M.Y., Zhang B., Zhao J.H. (2022). The Intestinal Microbiota and Metabolites in the Gut-Kidney-Heart Axis of Chronic Kidney Disease. Front. Pharmacol..

[184] Tong A.J., Li Z.Q., Liu X.Y., Ge X.D., Zhao R.F., Liu B., Zhao L.A., Zhao C. (2024). Laminaria japonica polysaccharide alleviates type 2 diabetes by regulating the microbiota-gut-liver axis: A multi-omics mechanistic analysis. Int. J. Biol. Macromol..

[185] Lin J.M., Wen J.M., Xiao N., Cai Y.T., Xiao J., Dai W.H., Chen J.P., Zeng K.W., Liu F.S., Du B. (2022). Anti-diabetic and gut microbiota modulation effects of sacha inchi (*Plukenetia volubilis* L.) leaf extract in streptozotocin-induced type 1 diabetic mice. J. Sci. Food Agric..

[186] Bai Z.Y., Huang X.J., Wu G.J., Ye H., Huang W.Q., Nie Q.X., Chen H.H., Yin J.Y., Chen Y., Nie S.P. (2023). Polysaccharides from red kidney bean alleviating hyperglycemia and hyperlipidemia in type 2 diabetic rats via gut microbiota and lipid metabolic modulation. Food Chem..

[187] Chen C., You L.J., Huang Q., Fu X., Zhang B., Liu R.H., Li C. (2018). Modulation of gut microbiota by mulberry fruit polysaccharide treatment of obese diabetic db/db mice. Food Funct..

[188] Ding W., Wang Y.N., Zhou J.F., Shi B. (2018). Effect of structure features of polysaccharides on properties of dialdehyde polysaccharide tanning agent. Carbohydr. Polym..

[189] Silva I.M.V., Machado F., Moreno M.J., Nunes C., Coimbra M.A., Coreta-Gomes F. (2021). Polysaccharide Structures and Their Hypocholesterolemic Potential. Molecules.

[190] Ji X.L., Guo J.H., Cao T.Z., Zhang T.T., Liu Y.Q., Yan Y.Z. (2023). Review on mechanisms and structure-activity relationship of hypoglycemic effects of polysaccharides from natural resources. Food Sci. Hum. Well.

[191] Ji N., Liu P., Zhang N., Yang S.Y., Zhang M.S. (2022). Comparison on Bioactivities and Characteristics of Polysaccharides from Four Varieties of Gastrodia elata Blume. Front. Chem..

[192] Wang H.T., Li H.L., Hou Y.T., Zhang P.J., Tan M.Q. (2023). Plant polysaccharides: Sources, structures, and antidiabetic effects. Curr. Opin. Food Sci..

[193] Dou Z.M., Chen C., Fu X. (2019). Digestive Property and Bioactivity of Blackberry Polysaccharides with Different Molecular Weights. J. Agric. Food Chem..

[194] Ferreira-Lazarte A., Kachrimanidou V., Villamiel M., Rastall R.A., Moreno F.J. (2018). In vitro fermentation properties of pectins and enzymatic-modified pectins obtained from different renewable bioresources. Carbohydr. Polym..

[195] Mao G.Z., Li S., Orfila C., Shen X.M., Zhou S.Y., Linhardt R.J., Ye X.Q., Chen S.G. (2019). Depolymerized RG-I-enriched pectin from citrus segment membranes modulates gut microbiota, increases SCFA production, and promotes the growth of Bifidobacterium spp., Lactobacillus spp. and Faecalibaculum spp.. Food Funct..

[196] Li Q., Li W.Z., Gao Q.Y., Zou Y.X. (2017). Hypoglycemic Effect of Chinese Yam (Dioscorea opposita rhizoma) Polysaccharide in Different Structure and Molecular Weight. J. Food Sci..

[197] Wei C.Y., Yao L., Zhang L., Zhang Y., Luo Q., Qiu S.Y., Zeng X.Y., Chen S.G., Ye X.Q. (2022). In Vitro Digestion and Fecal Fermentation of Peach Gum Polysaccharides with Different Molecular Weights and Their Impacts on Gut Microbiota. Foods.

[198] Lu X.Y., Xu H.T., Fang F., Liu J.C., Wu K.Z., Zhang Y.W., Wu J.H., Gao J. (2023). In vitro effects of two polysaccharide fractions from Laminaria japonica on gut microbiota and metabolome. Food Funct..

[199] Kim K.T., Rioux L.E., Turgeon S.L. (2014). Alpha-amylase and alpha-glucosidase inhibition is differentially modulated by fucoidan obtained from Fucus vesiculosus and Ascophyllum nodosum. Phytochemistry.

[200] Harris H.C., Edwards C.A., Morrison D.J. (2017). Impact of Glycosidic Bond Configuration on Short Chain Fatty Acid Production from Model Fermentable Carbohydrates by the Human Gut Microbiota. Nutrients.

[201] Fang F., Xiao C.Q., Wan C., Li Y.Q., Lu X.Y., Lin Y., Gao J. (2022). Two Laminaria japonica polysaccharides with distinct structure characterization affect gut microbiota and metabolites in hyperlipidemic mice differently. Food Res. Int..

